# *In vivo* CRISPRa decreases seizures and rescues cognitive deficits in a rodent model of epilepsy

**DOI:** 10.1093/brain/awaa045

**Published:** 2020-03-04

**Authors:** Gaia Colasante, Yichen Qiu, Luca Massimino, Claudia Di Berardino, Jonathan H Cornford, Albert Snowball, Mikail Weston, Steffan P Jones, Serena Giannelli, Andreas Lieb, Stephanie Schorge, Dimitri M Kullmann, Vania Broccoli, Gabriele Lignani

**Affiliations:** a1 San Raffaele Scientific Institute, Via Olgettina 58, 20132, Milan, Italy; a2 Department of Clinical and Experimental Epilepsy, UCL Institute of Neurology, University College London, London, UK; a3 Department of Pharmacology, UCL School of Pharmacy, University College London, London, UK; a4 CNR Institute of Neuroscience, Via Vanvitelli 32, 20129, Milan, Italy

**Keywords:** CRISPR, gene therapy, epilepsy, gene promoter, potassium channels

## Abstract

Epilepsy is a major health burden, calling for new mechanistic insights and therapies. CRISPR-mediated gene editing shows promise to cure genetic pathologies, although hitherto it has mostly been applied *ex vivo*. Its translational potential for treating non-genetic pathologies is still unexplored. Furthermore, neurological diseases represent an important challenge for the application of CRISPR, because of the need in many cases to manipulate gene function of neurons *in situ*. A variant of CRISPR, CRISPRa, offers the possibility to modulate the expression of endogenous genes by directly targeting their promoters. We asked if this strategy can effectively treat acquired focal epilepsy, focusing on ion channels because their manipulation is known be effective in changing network hyperactivity and hypersynchronziation. We applied a doxycycline-inducible CRISPRa technology to increase the expression of the potassium channel gene *Kcna1* (encoding K_v_1.1) in mouse hippocampal excitatory neurons. CRISPRa-mediated K_v_1.1 upregulation led to a substantial decrease in neuronal excitability. Continuous video-EEG telemetry showed that AAV9-mediated delivery of CRISPRa, upon doxycycline administration, decreased spontaneous generalized tonic-clonic seizures in a model of temporal lobe epilepsy, and rescued cognitive impairment and transcriptomic alterations associated with chronic epilepsy. The focal treatment minimizes concerns about off-target effects in other organs and brain areas. This study provides the proof-of-principle for a translational CRISPR-based approach to treat neurological diseases characterized by abnormal circuit excitability.

## Teaser

CRISPRa targets promoters to modulate gene activity. Colasante, Qiu *et al.* report that CRISPRa upregulation of an endogenous potassium channel reduces seizures and rescues cognitive defects in an epilepsy model. The study provides proof of principle for a translational CRISPR-based approach to treat neurological diseases.

## Introduction

Epilepsy affects up to 1% of the population, and 30% of patients continue to experience seizures despite the use of current medication (Kwan *et al.*, 2011; Tang *et al.*, 2017). Although the majority of drug-resistant epilepsies are focal, targeting drugs to a restricted brain region presents major challenges, and potentially curative surgery is limited to a minority of cases where the seizure focus is remote from eloquent cortex (Kullmann *et al.*, 2014). Gene therapy holds promise as a rational replacement for surgery for intractable pharmaco-resistant epilepsy, and could in principle improve the prospect for seizure freedom in many people (Kullmann *et al.*, 2014; Lieb *et al.*, 2018). Several approaches have been proposed to interfere with epileptogenesis or to decrease seizure frequency in chronic epilepsy (Simonato, 2014). Current experimental gene therapies mainly rely on viral vector-mediated expression of genes encoding normal CNS proteins or exogenous non-mammalian proteins (Richichi *et al.*, 2004; Noe *et al.*, 2008; Wykes *et al.*, 2012, 2016; Krook-Magnuson *et al.*, 2013; Kätzel *et al.*, 2014; Lieb *et al.*, 2018; Agostinho *et al.*, 2019). This approach has several potential limitations, including a finite packaging capacity of viral vectors, difficulty in ensuring normal splicing and post-transcriptional processing, and, in the case of non-mammalian membrane proteins, concerns about immunogenicity. Modulating the expression of endogenous genes, in contrast, would represent an important step towards safe and rational treatment of intractable epilepsy and other neurological diseases.

The DNA editor/regulator CRISPR/Cas9 (Konermann *et al.*, 2015; Dominguez *et al.*, 2016; Adli, 2018) represents a powerful tool to modify endogenous genes, not only in somatic cells but also in mammalian neurons (Heidenreich and Zhang, 2016; Suzuki *et al.*, 2016). In addition to permanently altering endogenous gene sequences, CRISPR/Cas9 can regulate the activity of genes through promoter modulation, an approach known as CRISPR activation (CRISPRa) (Dominguez *et al.*, 2016; Liao *et al.*, 2017; Matharu *et al.*, 2019). CRISPRa is therefore a promising tuneable tool to increase the expression of genes encoding, for instance, ion channels, in chronic epilepsy in order to restore physiological levels of network activity (Wykes *et al.*, 2012; Wykes and Lignani, 2018; Colasante *et al.*, 2020). The CRISPRa system consists of a nuclease-defective Cas9 (dCas9) fused to a transcription activator and a small guide RNA (sgRNA) that targets dCas9 to the promoter of the gene of interest (Dominguez *et al.*, 2016). There are multiple advantages of this system. First, it is versatile because the targeted gene can be switched simply by changing the sgRNA. Second, CRISPRa preserves the full range of native splice variants and protein biogenesis mechanisms (Liao *et al.*, 2017). Third, CRISPRa is, in principle, safe because it only alters the promoter activity of genes that are already transcribed in targeted neurons. Finally, CRISPRa can be targeted to specific neurons in the epileptic focus using established viral vectors (La Russa and Qi, 2015).

Here, we report the use of CRISPRa to treat a mouse model of temporal lobe epilepsy, from *in vitro* validation to demonstration of efficacy in reducing seizure frequency and rescuing cognitive impairment *in vivo*.

## Materials and methods

### Study design

This study aimed to test the hypothesis that upregulating endogenous genes (e.g. *Kcna1*) with CRISPRa can treat chemoconvulsant-induced temporal lobe epilepsy. The experiments were designed to achieve a power >0.8 with an α = 0.05. For *in vivo* experiments the 3Rs guidelines for animal welfare were followed. Outliers were not excluded and at least three independent repetitions were performed. Exclusion criteria were applied for all the recordings (see below). All the experiments were randomized and researchers were blinded during recordings and analysis.

### Animals and ethics

All experimental procedures were carried out in accordance with the UK Animals (Scientific Procedures) Act 1986. Male and female C57BL/6J and Camk2a-Cre mice (2–3 months old, 20–30 g, Envigo and Jackson Laboratory, respectively) were used for the experiments. Animals were housed in an enriched environment, in groups before surgery and singly after surgery in individually ventilated cages in a specific pathogen-free facility.

### Plasmids

Small guide RNAs (sgRNAs) were cloned into a lentiviral vector with a U6 promoter (pU6). Defective Cas9 fused to the VP160 activator domain was cloned into T2A with the Puromucin resistance cassette (Puro^R^) and under the control of the *Eef1a1* (Ef1alpha) promoter (Ef1alpha-dCas9VP160-T2A-Puro^R^). The dCas9VP160-2A-Puro^R^ cassette was obtained from pAC94-pmax-dCas9VP160-2A-Puro^R^ (a gift from R. Jaenisch) (Addgene plasmid #48226), and subcloned in a the TetO-FUW vector followed by restriction digestion with HpaI/AfeI, then blunt cloned into an Ef1alpha-GFP vector after GFP removal by SmaI/EcoRV digestion. Ef1alpha-dCas9VP160-T2A-GFP was obtained by restriction digestion of Ef1alpha-dCas9VP160-T2A-Puro^R^ with AscI/XbaI, which removed VP160-T2A-PuroR; the VP160-T2A fragment was then obtained by AscI/XhoI digestion from Ef1alpha-dCas9VP160-T2A-Puro^R^ while the GFP fragment was PCR amplified using primers containing XhoI/XbaI restriction sites; finally, the two fragments were ligated together into the vector. To obtain a single vector containing both dCas9A and sgRNA, the pU6-sgRNA cassette was HpaI digested and cloned into Ef1alpha-dCas9VP160. To generate an adeno-associated virus (AAV) with activating dCas9 (dCas9-VP64) under a doxycycline-inducible promoter, and tetracycline transactivator responsive element (TRE), we used AAV-SpCas9 (a gift from F. Zhang, Addgene #PX551) as the starting material: the *Mecp2* promoter was removed by XbaI/AgeI digestion and the TRE promoter was amplified using the primers: FWXbaI: 5′-GCTCTAGACCAGTTTGGTTAGATCTC-3′; and RV AgeI: 5′-GCACCGGTGCGATCTGACGGTTCACT-3′. SpCas9 was removed using AgeI/EcoRI and Cas9m4-VP64 (a gift from G. Church, Addgene #47319) was digested with AgeI/EcoRI. The VP64 fragment was PCR-amplified using the following primers with EcoRI sites: F: 5′-GATCATCGAGCAAATAAGCGAATTCTC-3′ and R: 5′-gctaaGAATTCTTA-TCTAGAGTTAATCAGCATG-3′. The AAV vector containing the sgRNA cassette was derived from pAAV-U6sgRNA (SapI)_hSyn-GFP-KASH-bGH (PX552 was a gift from F. Zhang, Addgene #60958): sg19 or lacZ were cloned under the U6 promoter and the GFP was removed by KpnI/ClaI digestion and replaced by a DIO-rtTA-T2A-Tomato cassette. This vector was used for the work in Camk2a-Cre mice. For the work in C57/Bl6 mice, this vector was XbaI/ClaI-digested to remove the human *SYN1* (hSyn) promoter, and the DIO-rtTA-T2A-Tomato cassette was replaced by a *Camk2a* promoter amplified with NheI-KpnI and a tTA T2a tomato cassette amplified with KpnI/ClaI, ligated together in the vector.

### Virus preparation

Lentiviruses were produced as previously described with a titre of 10^7^–10^8^ IU/ml (Colasante *et al.*, 2015). AAVs were produced as previously described with a titre >10^12^ vg/ml (Morabito *et al.*, 2017). The TRE-dCas9-VP64 AAV was produced by VectorBuilder with a titre of 8 × 10^12^ vg/ml.

### P19 cell line

P19 cells were cultured in alpha-MEM (Sigma-Aldrich) supplemented foetal bovine serum non-essential amino acids, sodium pyruvate, glutamine and penicillin/streptomycin and split every 2–3 days using 0.25% trypsin. For transfection, Lipofectamine™ 3000 (Thermo Fisher Scientific) was used according to the manufacturer’s instructions.

### Primary neuronal culture and lentivirus transduction

Cortical neurons were isolated from postnatal Day 0 C57Bl/6J mouse pups as previously described (Beaudoin *et al.*, 2012) and were transduced with lentiviruses at 1 day *in vitro* (DIV). Quantitative RT-PCR, RNA seq, western blot analysis and electrophysiology recordings were performed 14–16 days after transduction.

### RNA isolation and quantitative RT- PCR

RNA was extracted from primary neurons and cells using TRI Reagent^®^ (Sigma) according to the manufacturer’s instructions. For quantitative RT-PCR (RT-qPCR), cDNA synthesis was obtained using the ImProm-II™ Reverse Transcription System (Promega) and RT-qPCR was carried out with custom designed oligonucleotides (Supplementary Table 1) using the Titan HotTaq EvaGreen^®^ qPCR Mix (BIOATLAS). Analysis of relative expression was performed using the ΔΔC_T_ method, relative to the Ctrl-dCas9A condition. To determine *Kcna1* expression *in vivo* and for RNA-Seq, RNA was extracted from frozen tissues. For qPCR, ΔΔC_T_ was determined in Ctrl-dCas9A or in Kcna1-dCas9A injected hippocampi relative to contralateral hippocampi in epileptic animals at the end of the recordings.

### Western blot

Total neuronal protein extracts were obtained from the lysis of primary neurons by RIPA lysis buffer (150 mM NaCl, 1% Triton, 0.5% sodium deoxycholate, 0.1% sodium dodecyl sulphate, Tris pH 8.0 50 mM, protease inhibitor cocktail) 2 weeks after infection with the CRISPRa-Kcna1 system. Lysates were kept on ice for 30 min by vortexing every 10 min and then centrifuged at 4°C for 5 min at 5000 rpm. Supernatants with solubilized proteins were collected in new tubes and stored at −80°C until use. Western blot analysis was performed using primary antibodies against the following proteins: anti-K_v_1.1 (1:1000, NeuroMab) anti-α/βActin (1:10 000, Sigma).

### Bioinformatic analysis

Encyclopedia of DNA Elements (ENCODE) and the Functional ANnoTation Of the Mammalian genome (FANTOM) (Carninci *et al.*, 2006) databases were used to download transcriptomics and epigenetics NGS data. Tracks were visualized along the mm10 mouse reference genome with the Integrative Genome Viewer (IGV) (Thorvaldsdottir *et al.*, 2013).

### Off-targets

Using the Galaxy web-tool (https://usegalaxy.org/) we generated two datasets: one containing sg19 off-target sequences predicted by the *CRISPOR* web tool (http://crispor.tefor.net) and one containing all the 500-bp genomic regions (NCBI37/mm9) upstream to transcription start sites of annotated transcripts. Intersecting the two datasets, all sg19 off-target sequences in putative gene promoters were derived. To identify genes regulated by putative promoters, the sequence of the predicted off-targets was aligned by IGV to the reference genome and to transcripts annotated in ENSEMBL database. Validation of expression levels of putative off-target genes was performed by RT-qPCR.

### RNA-Seq

RNA libraries for both *in vitro* and *in vivo* experiments were generated starting from 1 μg of total RNA extracted from sglacz- and sg19-dCas9A neurons at 10 DIV. RNA quality was assessed using a TapeStation instrument (Agilent) and only RNA samples with integrity number (RIN) ≥8 were analysed. For *in vitro* experiments, RNA was processed according to the Lexogen QuantSeq 3’ mRNA-Seq Library Prep Kit protocol and the libraries were sequenced on an Illumina NextSeq 500 with 75-bp stranded reads at CTGB, Ospedale San Raffaele. Fastq files were aligned to the mouse genome (NCBI37/mm9) with Bowtie2.

For *in vivo* experiments, RNA was processed according to the TruSeq Stranded mRNA Library Prep Kit protocol. The libraries were sequenced on an Illumina HiSeq 3000 with 76 bp stranded reads using Illumina TruSeq technology at Genewiz. Image processing and base calling were performed using the Illumina Real Time Analysis Software. Fastq files were mapped to the mm10 mouse reference genome with the STAR aligner v2.7 (Dobin *et al.*, 2013).

Differential gene expression and functional enrichment analyses were performed with DESeq2 (Love *et al.*, 2014) and GSEA, respectively. Statistical and downstream bioinformatics analyses were performed within the R environment. Gene expression heat maps were produced with GENE-E (Broad Institute). Data of *in vitro* and *in vivo* experiments were deposited in the NCBI Gene Expression Omnibus repository with the GSE133930 GEO ID.

### Slice preparation

Camk2a-Cre mice of either sex (2–3 months old) were sacrificed by cervical dislocation under isoflurane. Brains were quickly dissected into ice-cold oxygenated slicing solution (in mM: 75 sucrose, 2.5 KCl, 25 NaHCO_3_, 25 glucose, 7 MgCl_2_, 0.5 CaCl_2_) and cut into 300 µm coronal slices using a Leica VT1200S vibratome (Leica). Slices were stored submerged in oxygenated recording artificial CSF (in mM: 25 glucose, 125 NaCl, 2.5 KCl, 25 NaHCO_3_, 1 MgCl_2_, 1.25 NaH_2_PO_4_.H_2_O and 2 CaCl_2_) at 32°C for 30 min and at room temperature for a further 30 min before recording.

### Electrophysiology

#### In vitro

For current-clamp recordings, the internal solution contained (in mM): 126 K-gluconate, 4 NaCl, 1 MgSO_4_, 0.02 CaCl_2_, 0.1 BAPTA, 15 glucose, 5 HEPES, 3 ATP-Na2, 0.1 GTP-Na, pH 7.3. The extracellular (bath) solution contained (in mM): 2 CaCl_2_, 140 NaCl, 1 MgCl_2_, 10 HEPES, 4 KCl, 10 glucose, pH 7.3. d-(−)-2-amino-5-phosphonopentanoic acid (D-AP5; 50 μM), 6-cyano-7-nitroquinoxaline-2,3-dione (CNQX; 10 μM) and picrotoxin (PTX; 30 μM) were added to block synaptic transmission. Transduced excitatory neurons were identified with EGFP fluorescence and from a pyramidal somatic shape. Neurons with unstable resting potential (or >−50 mV), access resistance (R_a_) >15 MΩ and/or holding current >200 pA at −70 mV were discarded. Bridge balance compensation was applied and the resting membrane potential was held at −70 mV. A current step protocol was used to evoke action potentials by injecting 250-ms long depolarizing current steps of increasing amplitude from −20 pA (Δ10 pA). Recordings were acquired using a MultiClamp™ 700A amplifier (Axon Instruments, Molecular Devices) and a Power3 1401 (CED) interface combined with Signal software (CED), filtered at 10 kHz and digitized at 50 kHz.

#### *Ex vivo* current clamp recordings

Current clamp recordings were performed in standard external solution in the presence of DL-AP5 (50 μM), CNQX (10 μM) and PTX (30 μM) to block NMDA, AMPA/kainate, and GABA_A_ receptors, respectively. The internal solution was the same as for *in vitro* patch clamp recordings. Neurons with holding current >100 pA and R_a_ >20 MΩ upon whole-cell break-in in voltage clamp mode and membrane potential less negative than −60 mV in current clamp were not considered for analysis. A 1440 Digidata^®^ (Molecular Devices) or Power3 1401 (CED) interface and MultiClamp™ 700A (Molecular Devices) amplifier were used.

#### *In vitro* and *ex vivo* electrophysiology analysis

All the electrophysiology analysis was performed with an automated Python script. Passive properties were calculated from the hyperpolarizing steps of the current clamp steps protocol. Input resistance was averaged from three current steps (two negative and one positive). Capacitance was calculated from the hyperpolarizing current step as follows. First, the input resistance was determined as the steady state ΔV/ΔI (voltage/current), then the cell time constant (tau) was obtained by fitting the voltage relaxation between the baseline and the hyperpolarizing plateau. Capacitance was then calculated as tau/resistance. Single action potential parameters were calculated as previously described (Pozzi *et al.*, 2013). An event was detected as an action potential if it crossed 0 mV and if the rising slope was >20 mV/ms in a range of injected currents from 0 pA to 500 pA. All the experiments were performed at room temperature (22–24°C). All recordings and analyses were carried out blind to vector transduced.

#### Activity clamp

The template simulating the barrage of synaptic conductances during epileptiform bursts was previously described (Morris *et al.*, 2017). Dynamic clamp software (Signal 6.0, Cambridge Electronic Design, Cambridge, UK) and a Power3 1401 (CED) were used to inject both excitatory and inhibitory conductance templates simultaneously in a neuron recorded in current clamp configuration (iteration frequency 15 kHz). E_rev_ was set to 0 mV and −75 mV for excitatory and inhibitory conductances, respectively, and corrected for a liquid junction potential of 14.9 mV. Incrementing synaptic conductances were injected in recorded neurons to establish the conductance threshold for action potential generation. Current clamp recordings for activity clamp were performed with the same external and internal solutions as given above.

### Surgical procedures

All surgical procedures were performed in anaesthetized adult mice (2–3 months) placed in a stereotaxic frame (Kopf).

#### Epilepsy model

Kainic acid (0.3 µg of 10 mg/ml, Tocris) was injected in a volume of 200 nl (7.14 mM effective concentration) in the right amygdala (antero-posterior: −0.94; medio-lateral: 2.85; dorso-ventral: 3.75) at 200 nl/min under isoflurane anaesthesia (surgery time 10–15 min). The mice were allowed to recover from anaesthesia at 32°C for 5 min and then moved back to their cage where they were monitored closely during status epilepticus. Status epilepticus (characterized by stage 5 seizures on the Racine scale) usually began 10–15 min after complete recovery and always ended 40 min after kainic acid injection with 10 mg/kg intraperitoneal diazepam. Only animals that exhibited at least one seizure per week were included in the subsequent study.

#### Stereotaxic viral injection

AAV9 viruses (300 nl, 1:1 ratio) were injected with a 5-µl Hamilton syringe (33-Gauge) at 100 nl/min in three different coordinates of the right ventral hippocampus (Paxinos Mouse Brain Atlas; antero-posterior: −2/3 bregma/lambda distance; medio-lateral: −3; dorso-ventral: 3.5/3/2.5). The needle was kept in place for 10 min after each injection.

#### Transmitter implantation

An electrocorticogram (ECoG) transmitter (A3028C-CC Open Source Instruments, Inc.) was subcutaneously implanted and the recording electrode was placed in the cortex above the viral injection site (antero-posterior: −2/3 bregma/lamda distance; medio-lateral: −3). The ground electrode was placed in the contralateral frontal hemisphere.

#### Doxycycline diet

Animal food was changed to doxycycline *ad libitum* pellet (TD.120769-BLUE 625 mg/kg) after baseline recordings for the following 2 weeks.

#### Exclusion criteria

Only animals recorded for the entire period of the experiment (6 weeks after kainic acid) were used in the analysis. At the end of the experiments some animal tissues were analysed with qRT-PCR and others were verified with immunofluorescence. Of the 42 mice injected with kainic acid, 34 (80%) were implanted and injected, 20% either did not reach stage 5 of the Racine scale or were excluded because of infections or unexpected death in the first few days before planned implantation. Twenty-four mice were recorded for the entire duration of the experiment. Ten animals were not included in the analysis because they either died before the end of the recordings (*n *=* *2) or were culled because of technical issues (detachment of the headpiece) or transmitter failure. Two did not express dCas9 and were therefore excluded from the analysis. Twenty-two mice were used for the analysis (13 Ctrl-dCas9A and nine Kcna1-dCas9A). To avoid possible bias, all exclusions were made while researchers were blinded to treatment.

#### Pilocarpine acute seizure model

Male wild-type C57BLC/6J mice (3 months old) were anaesthetized with isoflurane and placed in a stereotaxic frame (David Kopf Instruments Ltd.). The animals were injected with 1.5 µl AAV CamKII-CRISPR-Kcna1/CamKII-CRISPR-LacZ at 100 nl/min in layer 2/3–5 primary visual cortex (coordinates: antero-posterior −2.8 mm, medio-lateral 2.4 from the bregma, and dorso-ventral 0.7/0.5/0.3 from pia). For ECoG monitoring, the recording electrode of 256 Hz single-channel ECoG transmitter (A3028C-CC, Open Source Instruments Inc.) was placed at the same coordinates. A reference electrode was placed in the contralateral skull. A cannula (Bilaney Consultants Ltd.) was implanted in the same location as the recording electrode for sequential pilocarpine injections. Animals were allowed to recover for 2 weeks before induction of acute seizures by pilocarpine (3.5 M in saline) (Magloire *et al.*, 2019) injected 0.5 mm below the cannula using a microinjection pump (WPI Ltd.), a 5-µl Hamilton syringe (Esslab Ltd.), and a 33-Gauge needle (Esslab Ltd.). The injection volume was incremented on consecutive days (180 nl, 300 nl and 500 nl) until spike-wave discharges were observed, and recorded as the threshold dose. If seizures failed to terminate spontaneously, the animal was excluded from the study. To assess the treatment, the animals were placed on a doxycycline diet for 7 days and only the threshold dose for the animal was repeated. ECoG monitoring was used to assess seizure severity for an hour after the pilocarpine injection. The researcher who acquired and analysed the data was blinded to the virus injected.

### EEG (or ECoG) recordings

The ECoG was acquired wirelessly using hardware and software from Open Source Instruments, Inc. The ECoG was sampled at a frequency of 256 Hz, band-pass filtered between 1 and 160 Hz, and recorded continuously for the duration of the experiments. The animals were housed independently in a Faraday cage.

### EEG analysis

Spontaneous seizures were detected from chronic recordings using a semi-automated supervised learning approach (Supplementary Fig. 7). First, a library containing examples of epileptiform activity was built using seizures identified from visual inspection of ECoG data. The recordings were saved in hour-long files, and for each seizure this full hour was included in the library. Recordings were chunked into 5-s blocks that were labelled as either ‘ictal’ or ‘interictal’ if they contained epileptiform-labelled activity or not, respectively. For each 5-s chunk of recording, 15 features were extracted (Supplementary Fig. 7 and see online resource below). A random forest discriminative classifier was trained on the features and labels of each of the 5-s examples in the library (Breiman, 2001). In addition, cross-validation generated classifier predictions were used to parameterize a Hidden Markov Model in which the hidden states were the human annotations and the emissions the classifier predictions. For automated detection of epileptiform activity from unlabelled recordings, the discriminative classifier was first used to predict the class of consecutive 5-s chunks. We then applied the forward-backward algorithm to obtain the marginal probability of being in seizure state for each recording chunk given the surrounding classifier predictions. The smoothed predictions were then manually verified, false positives removed from the analysis and start and end locations adjusted. To quantify the performance of our approach, we randomly selected four 2-week sets of recordings and visually examined the traces for seizures and compared them to classifier predictions (blinded). During the 8 weeks, we did not visually detect any seizures that were not marked by the classifier—as such, for this model of epilepsy, our false negative rate was <1/300. False positives were less of a concern, but in general we observed ≪ *n* seizures for a given period of time. For further information and code, see: https://github.com/jcornford/pyecog.

### Video recordings

IP cameras from Microseven (https://www.microseven.com/index.html) were used and synchronized via the Windows time server to the same machine as used to acquire the ECoG. Continuous video recordings produced six videos per hour.

### Immunohistochemistry

Immunostaining was performed on 50-µm mouse brain PFA-fixed sections with the following antibodies: mouse anti-GAD67 (MAB5406, Merck), rabbit anti-RFP (600-401-379, Rockland), Alexa Fluor^®^ 555 goat anti-rabbit (A32732, Invitrogen) and Alexa Fluor^®^ 488 goat anti-mouse (A32723, Invitrogen). Images were acquired with ZEN software (Zeiss) on a LSM710 confocal microscope (Zeiss) and co-localization analysis of tdTomato and GAD67 were performed with ImageJ 1.51n (Wayne Rasband, National Institute of Health) plugin ‘JACoP’.

### Behaviour tests

Trials started 2 weeks post-virus injection, all were carried out between 7 am and 7 pm, during the light phase. Animals (3 months old) were habituated in the designated behaviour room for at least 15 min in home cages prior to the test.

### Object Location Test

For the familiarization phase, mice were placed individually in the arena (50 cm × 50 cm × 40 cm) and for 8 min, were allowed to explore two identical objects placed in the arena at least 5 cm away from the border. After a 6-h retention delay, the animals were returned to the same arena with one of the objects randomly relocated to a new location. The animal was allowed to explore for 8 min with video recordings. The arena and objects were thoroughly cleaned with ethanol between each session.

### Novel Object Recognition Test

Twenty-four hours after the Object Location Test, the same animals were subjected to the Novel Object Recognition Test. The familiarization session was the same as for the Object Location Test. After a 6-h retention delay, one of the objects was randomly replaced by a novel object with a different shape and surface texture. The animals were allowed to explore freely for 8 min (Leger *et al.*, 2013).

All trials were recorded with a Raspberry Pi 3B+ equipped with a V1 camera module (https://www.raspberrypi.org/documentation/hardware/camera/) and using Raspivid version 1.3.12 as 1296 × 972 pixel, 30 frame/s MP4 video files. Automated analysis was carried out with custom scripts written in Bonsai version 2.4-preview (Lopes *et al.*, 2015). A researcher blinded to the treatment assessed and scored the exploration time manually after automated analysis. Discrimination index (DI) was calculated using the following formula: (time spent with altered object − time spent with unchanged object) / (total time spent exploring objects).

### Statistical analysis

Data are plotted as box and whiskers, representing interquartile range (box), median (horizontal line), and maximum and minimum (whiskers), together with all the points. The mean is further shown as a plus symbol. The statistical analysis performed is shown in each figure legend. Deviation from normal distributions was assessed using D’Agostino-Pearson’s test, and the *F*-test was used to compare variances between two sample groups. Student’s two-tailed *t-*test (parametric) or the Mann-Whitney test (non-parametric) were used as appropriate to compare means and medians. Fisher’s exact test was used to analyse the contingency table. To compare two groups at different time points we used two-way repeated measure ANOVA, followed by Bonferroni *post hoc* test for functional analysis. Statistical analysis was carried out using Prism (GraphPad Software, Inc., CA, USA) and SPSS (IBM SPSS statistics, NY, USA).

### Data availability

All the Python and Bonsai scripts are freely available. Plasmids will be deposited with Addgene, and transcriptomic data are deposited in the NCBI Gene Expression Omnibus repository with the GSE133930 GEO ID.

## Results

### CRISPRa targeting the *Kcna1* promoter region increases K_v_1.1 and decreases neuronal excitability

**Figure 1:**
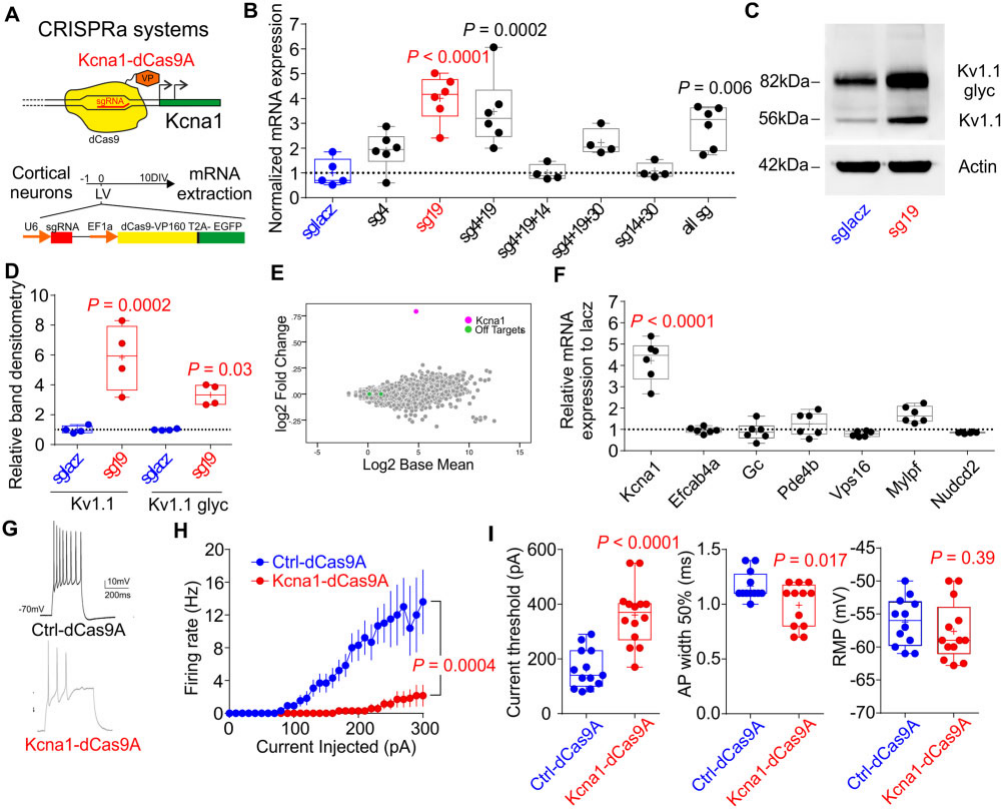
**CRISPRa increases endogenous *Kcna1* expression reducing neuronal excitability *in vitro*.** (**A**) Schematic representation of the CRISPRa approach to increasing *Kcna1.* Primary neurons were transduced with lentivirus 1 day after plating and experiments were performed after 10 days for qPCR and western blotting, and 14 days for electrophysiology. (**B**) *Kcna1* mRNA expression normalized to the control LacZ sgRNA (blue) for the most effective sgRNAs and combinations of different sgRNAs tested in P19 cells (Supplementary Fig. 1). One-way ANOVA followed by Bonferroni multiple comparison test versus sgLacZ. (**C** and **D**) Western blots were used to determine the increase in K_v_1.1 and glycosylated K_v_1.1 (glyc) in neurons transduced either with dCas9A and sg19 (red), or sgLacZ (blue). Student’s *t*-test. (**E**) MA plots showing log_2_ fold-change as a function of log_2_ base mean expression of *Kcna1*-dCas9A treated neurons with respect to Ctrl-dCas9A. *Kcna1 *=* *pink; off-targets = green; all other genes = grey. (**F**) Messenger RNA expression relative to sgLacZ for each off-target. The expression level of sgLacZ is represented as the dashed line at 1. Multiple Student’s *t*-tests, each compared to control and corrected for multiple comparison (α = 0.0083). (**G**) Representative traces of recordings from neurons transduced either with Ctrl-dCas9A (sgLacZ, blue) or Kcna1-dCas9A (sg19, red) and injected with 300 pA steps in current clamp. (**H**) Average firing rates in response to different current injections for neurons transduced with ctrl-dCas9A or Kcna1-dCas9A. Two-way RM ANOVA. (**I**) Neuronal and action potential (AP) properties in neurons transduced with ctrl-dCas9A or Kcna1-dCas9A. Student’s *t*-test.

We first asked if CRISPRa can be exploited to increase endogenous gene expression in glutamatergic neurons and decrease their excitability. As a proof-of-principle, we chose the *Kcna1* gene encoding the K_v_1.1 channel, which is important for the regulation of neuronal action potential firing and synaptic transmission (Pinatel *et al.*, 2017; Vivekananda *et al.*, 2017). Lentivirus- or AAV-mediated overexpression of *Kcna1* reduces neuronal excitability and, when targeted to principal cells, suppresses seizures in rodent models of epilepsy (Wykes *et al.*, 2012; Snowball *et al.*, 2019). We first conducted a bioinformatic analysis to identify its promoter region. Alignment of datasets of gene expression and epigenetic markers of actively transcribed genes in perinatal and adult mouse brain identified peaks of enrichment for RNA PolII, mono- and tri-methylation of Lys4 and acetylation of Lys27 of H3 histone along the gene (Supplementary Fig. 1A). One of these regions was located immediately upstream to the annotated *Kcna1* transcription start site and identified as a suitable target for CRISPRa. We submitted 200 bp from this region to the *CRISPOR* web tool for sgRNA design, and selected four candidate guides (sg4, sg14, sg19 and sg30) for validation, initially in the mouse P19 cell line that expresses many neuronal genes. SgRNAs were lipofected individually or in combination, together with a construct carrying dCas9 fused to the transcriptional activator VP160 (dCas9-VP160) and a puromycin resistance cassette. dCas9-VP160 with sg4, sg14 or sg19, but not with sg30, significantly upregulated the expression of the *Kcna1* gene. We focused on sg4 and sg19, which induced the highest levels of *Kcna1* expression (Supplementary Fig. 1B). When tested in combination, sg4 and sg19 together were also efficacious, but not sg4 and sg30 (Supplementary Fig. 1B). We confirmed that the highest efficiency of upregulation of *Kcna1* in primary neurons was achieved with sg19 (Fig. 1A and B). Consequently, we generated a construct carrying dCas9-VP160 driven by the Ef1α promoter and either the sg19 targeting the *Kcna1* promoter (Kcna1-dCas9A) or a control sgRNA targeting LacZ (Ctrl-dCas9A). Western blot analysis confirmed increased K_v_1.1 protein levels in sg19-treated neurons when compared to the sgLacZ control. Importantly, we detected increased levels of glycosylated K_v_1.1, corresponding to mature protein, implying normal processing of the upregulated potassium channel (Fig. 1C and D) (Watanabe *et al.*, 2003).

The *CRISPOR* tool predicted 250 putative off-target genes for sg19, mostly with a very low likelihood score. To evaluate the specificity of CRISPRa we performed a gene expression profile analysis in primary neurons treated with Kcna1-dCas9A and compared this with Ctrl-dCas9A transduced neurons. No consistent alteration in the transcriptome of sg19 treated neurons was observed, except for a significant increase in *Kcna1* (Fig. 1E, red dot). Six of 250 predicted off-target genes for sg19 were located close to promoters of *Mylpf*, *Efcab4a, Nudcd2*, *Pde4b*, *Gc* and *Vps16* genes. However, none of these genes showed a significant change in expression in either the transcriptome analysis (Fig. 1E, green dot) or in quantitative RT-PCR assays (Fig. 1F).

Exogenous *Kcna1* overexpression results in a decrease in neuronal excitability and neurotransmitter release (Heeroma *et al.*, 2009; Wykes *et al.*, 2012). To test the functional efficacy of the CRISPRa system, primary neurons were transduced at 1 DIV with a lentivirus expressing Kcna1-dCas9A or Ctrl-dCas9A. After 14–16 DIV we used whole-cell patch clamp recordings to analyse neuronal excitability of both experimental groups (Fig. 1G). The maximal firing frequency was significantly decreased in neurons transduced with Kcna1-dCas9A when compared to Ctrl-dCas9A (Fig. 1H). Other excitability parameters sensitive to K_v_1.1 were also changed in neurons transduced with Kcna1-dCas9A in comparison with Ctrl-dCas9A: the current threshold was increased, and action potential width was decreased (Fig. 1I). However, passive membrane properties and other action potential properties were unchanged apart from a decrease in the input resistance (Supplementary Fig. 2). This change suggests that the effect of expression of the endogenous promoter using CRISPRa is different than overexpression using virally delivered cDNA (Heeroma *et al.*, 2009).

### Kcna1-dCas9A decreases CA1 pyramidal cell excitability

**Figure 2:**
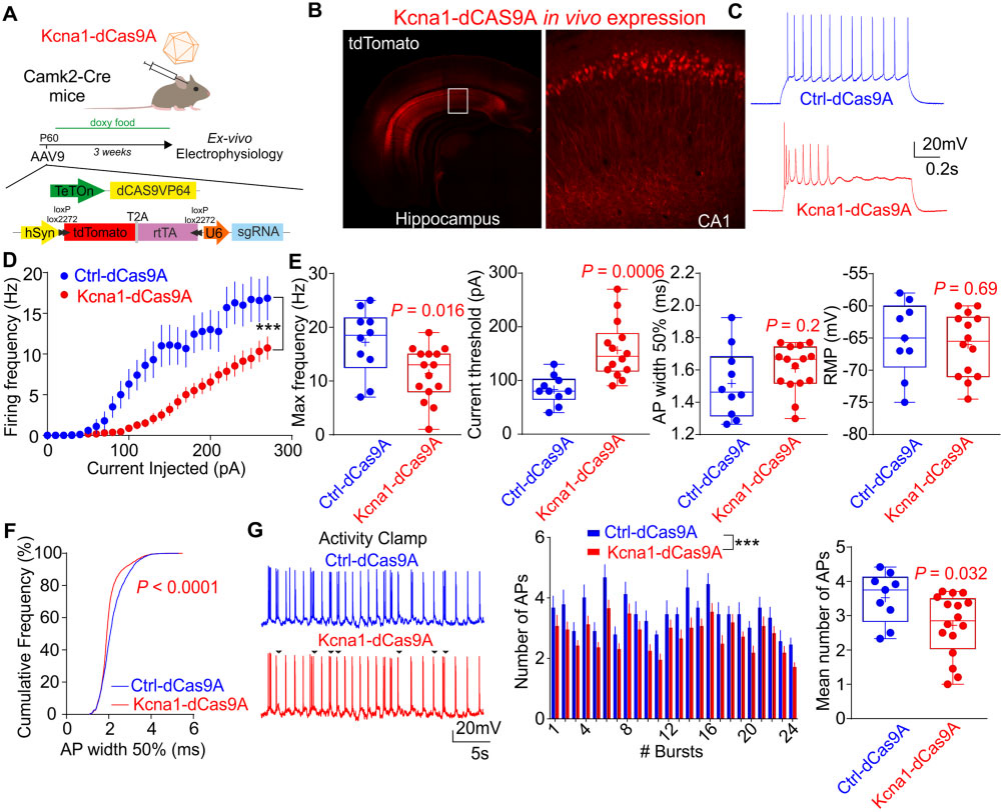
**CRISPRa delivered with AAV9 increases endogenous *Kcna1* expression and reduces CA1 pyramidal neuron excitability.** (**A**) Schematic representation of the approach for *ex vivo* quantification of CRISPRa effects. Mice were injected with AAVs at approximately postnatal Day (P)60 and experiments were performed 3 weeks after. (**B**) Representative live image of *Kcna1-*dCas9A expression in the hippocampus (bregma −2.70; *inset* = CA1 region, red = tdTomato) (**C**) Representative traces from CA1 pyramidal neurons, transduced either with Ctrl-dCas9A (sgLacZ, blue) or Kcna1-dCas9A (sg19, red) in pryramidal neurons injected with 280 pA steps in current clamp. (**D**) Average firing rates in response to different current steps for neurons transduced with Ctrl-dCas9A or Kcna1-dCas9A. Two-way repeated measures ANOVA. (**E**) Neuronal and action potential (AP) properties in neurons transduced with Ctrl-dCas9A or Kcna1-dCas9A. Maximal firing rate, current threshold to elicit the first action potential, action potential width and resting membrane potential are shown. Student’s *t*-test. (**F**) Cumulative frequency (%) of the action potential widths in neurons injected with 280 pA of current. Kolmogorov-Smirnov test for cumulative distributions. (**G**) Activity clamp protocol to mimic 24 interictal bursts of synaptic input from an epileptic network in neurons transduced with Ctrl-dCas9A or Kcna1-dCas9A (*left*). Black arrows represent the action potentials missed in the Kcna1-dCas9A neurons. Number of action potentials for each burst showed as mean ± standard error of the mean (SEM) (*middle*). Two-way ANOVA. The histogram represents the average number of action potentials for each neuron in the 24 bursts (*right*). Student’s *t*-test.

To test the efficacy of CRISPRa *in vivo*, we subcloned the CRISPRa elements in two separate AAV9 viral vectors that spread more efficiently than lentiviruses in the brain parenchyma. One AAV vector carried the dCas9-VP64 under the control of a TRE, while the other vector included the sg19 (or sgLacZ as a control) element and, to confer specific neuronal expression, a human synapsin promoter (hSyn) upstream to an inverted rtTA-t2a-tdTomato cassette flanked by loxP and lox511 sites. The separation of the CRISPRa tool into two constructs, and the choice of dCas9-VP64 over dCas9VP160, was dictated by the reduced cargo capacity of AAV vectors. Nevertheless, the dual AAV9 system has been shown to give a good level of neuron co-transduction (Sun *et al.*, 2019). This experimental design allowed the Kcna1-dCas9A system to be activated in forebrain principal neurons of Camk2a-cre mice transduced with both AAVs, but only after doxycycline administration (Fig. 2A and B). We co-injected both AAVs in the hippocampus of 2–3-month-old Camk2a-Cre mice, which were subsequently fed a doxycycline diet for 3 weeks before collecting acute brain slices for analysis. Pyramidal neurons in the CA1-subiculum of the ventral hippocampus were recorded with whole-cell patch clamp to measure their excitability (Fig. 2B, C and Supplementary Figs 3 and 4). Consistent with data from primary cultures, neurons transduced with Kcna1-dCas9A showed a decreased firing rate and increased current threshold when compared with Ctrl-dCas9A expressing neurons (Fig. 2D and E). A minor difference from primary cortical cultures was that the half-width of the first spike was not significantly different between Kcna1-dCas9A- and Ctrl-dCas9A-expressing neurons (Fig. 1I). However, a significant decrease in half-width was seen when all the action potentials during the current step protocol were pooled (Fig. 2F). Finally, we applied activity clamp, a method to assess neuronal excitability during epileptiform barrages of excitation (Morris *et al.*, 2017). Neurons expressing Kcna1-dCas9A fired less than neurons expressing Ctrl-dCas9A when exposed to the same simulated synaptic input. Taken together, these results support using Kcna1-dCas9A as a candidate antiseizure gene therapy (Fig. 2G).

### Kcna1-dCas9A decreases seizure frequency in a mouse model of temporal lobe epilepsy

**Figure 3:**
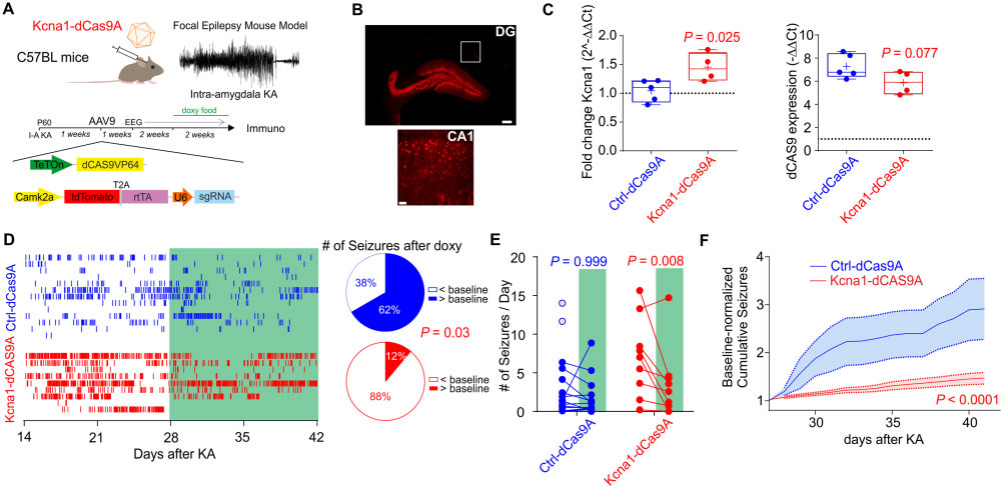
**CRISPRa-Kcna1 decreases number of seizures in a mouse model of acquired intractable temporal lobe epilepsy.** (**A**) Schematic representation of the CRISPRa approach used *in vivo* to treat the intra-amygdala kainic acid (KA) focal model of temporal lobe epilepsy. Mice were injected with AAVs 1 week after kainic acid treatment. Baseline EEG recordings were started the following week and continued for 2 weeks. Two further weeks of EEG recordings were performed after doxycycline food supplementation. (**B**) Representative immunofluorescence 7 weeks after status epilepticus of neurons transduced with Ctrl-dCas9A 4 weeks after status epilepticus. Scale bars: DG = 250 μm; CA1 = 50 μm. (**C**) Quantitative PCR analysis of *Kcna1* and dCas9 expression in the ipsilateral hippocampus relative to the contralateral hippocampus in epileptic mice transduced with either Ctrl-dCas9A or Kcna1-dCas9A at the end of the experiments. Student’s *t*-test. (**D**) *Left:* Raster plot showing all seizures before and after doxycycline administration in 13 mice treated with Ctrl-dCas9A and nine mice treated with Kcna1-dCas9A. *Right:* Pie charts showing the proportion of animals showing either more or fewer seizures after doxycycline food than during the baseline. Fisher’s exact test. (**E**) Number of seizures/day before and after doxycycline administration in control-dCas9 (*n *=* *13) and Kcna1-dCas9 (*n *=* *9) treated animals. Two-way ANOVA followed by Bonferroni multiple comparison test. Empty blue circles are animals that died during baseline and excluded from the analysis. (**F**) Cumulative plot of seizures normalized to the baseline in mice transduced with either ctrl-dCas9A or Kcna1-dCas9A. Two-way ANOVA.

We administered Kcna1-dCas9A in a mouse model of acquired epilepsy. C57BL/6J wild-type animals were injected with kainic acid in the right amygdala (Jimenez-Mateos *et al.*, 2012). This induced a period of status epilepticus, which was quantified by video recording to monitor seizure severity (Supplementary Fig. 5 and Supplementary Video 1). One week later, we injected either Kcna1-dCas9A or Ctrl-dCas9A AAVs in the right ventral hippocampus, and at the same time we implanted wireless EEG transmitters (bandwidth 1–256 Hz). For these experiments an AAV9 carrying a rtTA-t2a-tdTomato cassette without flanking recombination sites but driven by a *Camk2a* promoter was delivered to bias expression towards excitatory neurons. After a week of recovery to allow expression of the constructs, we began continuous video-EEG recording for 2 weeks (baseline) and then continued recording for two further weeks with doxycycline administration (Fig. 3A). Immunohistochemistry of the injected hippocampi showed expression of the tdTomato reporter in dentate gyrus granule cells and hippocampal CA3 excitatory neurons, as well as CA1 pyramidal cells (Fig. 3B and Supplementary Fig. 6). We extracted both the ipsilateral and contralateral hippocampi of 11 mice after the EEG recordings to analyse *Kcna1* and dCas9 expression. Injected hippocampi from two mice failed to express dCas9 and were excluded from the analysis. The injected hippocampi from the remaining nine mice (five Ctrl-dCas9A and four Kcna1-dCas9A) expressed dCas9 and showed a 50% increase in *Kcna1* expression in mice transduced with Kcna1-dCas9A compared to the contralateral counterpart, while no difference was detected in Ctrl-dCas9A hippocampi (Fig. 3C).

To investigate the ability of Kcna1-dCas9 to treat chronic temporal lobe epilepsy we analysed the frequency of generalized tonic-clonic seizures (Racine stage 5) in each animal before and after doxycycline administration using continuous video-EEG recordings (Fig. 3D–F, Supplementary Fig. 7 and Supplementary Video 2). Animals treated with Kcna1-dCas9A showed a significant reduction in the number of seizures after doxycycline was added to the food (Fig. 3D–F). The number of seizures after doxycycline administration decreased in eight of nine animals treated with Kcna1-dCas9A compared to only 5 of 13 animals treated with Ctrl-dCas9 (Fig. 3D). Only Kcna1-dCas9A treated animals showed a significant decrease in the number of seizures per day after doxycycline administration (Fig. 3E and F). There was no significant difference in seizure frequencies during the baseline period prior to doxycycline administration between the animals treated either with Kcna1-dCas9A or Ctrl-dCas9A (Fig. 3E, *P *=* *0.09 two-way ANOVA followed by Bonferroni multiple comparison test). However, two Ctrl-dCas9A animals, but none of the Kcna1-dCas9A animals, died during the baseline period from severe seizures and were not included in the comparison of seizure frequencies before and after doxycycline (Fig. 3E). We cannot exclude a protective effect of a low level of basal activation of CRISPRa in the Kcna1-dCas9A animals. Other EEG parameters, such as broadband power, seizure duration and EEG power for night-time and day-time periods were not changed by the treatment (Supplementary Figs 5 and 7). Taken together, these results show that Kcna1-dCas9A reduces the probability of tonic-clonic seizure initiation, but otherwise does not change seizure properties recorded in the cortex.

We complemented the chronic epilepsy study by looking at the effect of Kcna1-dCas9A treatment on acute seizures evoked by a different mechanism, focal pilocarpine injection in the visual cortex before and after doxycycline administration (Lieb *et al.*, 2018; Magloire *et al.*, 2019). We did not observe robust differences in EEG coastline and power in different frequency bands recorded for 1 h after pilocarpine in either group, although there was a non-significant trend for Kcna1-dCas9A animals to show an attenuation in seizure severity (Supplementary Fig. 8). These data argue that the action of Kcna1-dCas9A on experimental epilepsy goes beyond an effect on individual seizures.

### Kcna1-dCas9A rescues cognitive deficits and mitigates dysregulation of gene expression

**Figure 4:**
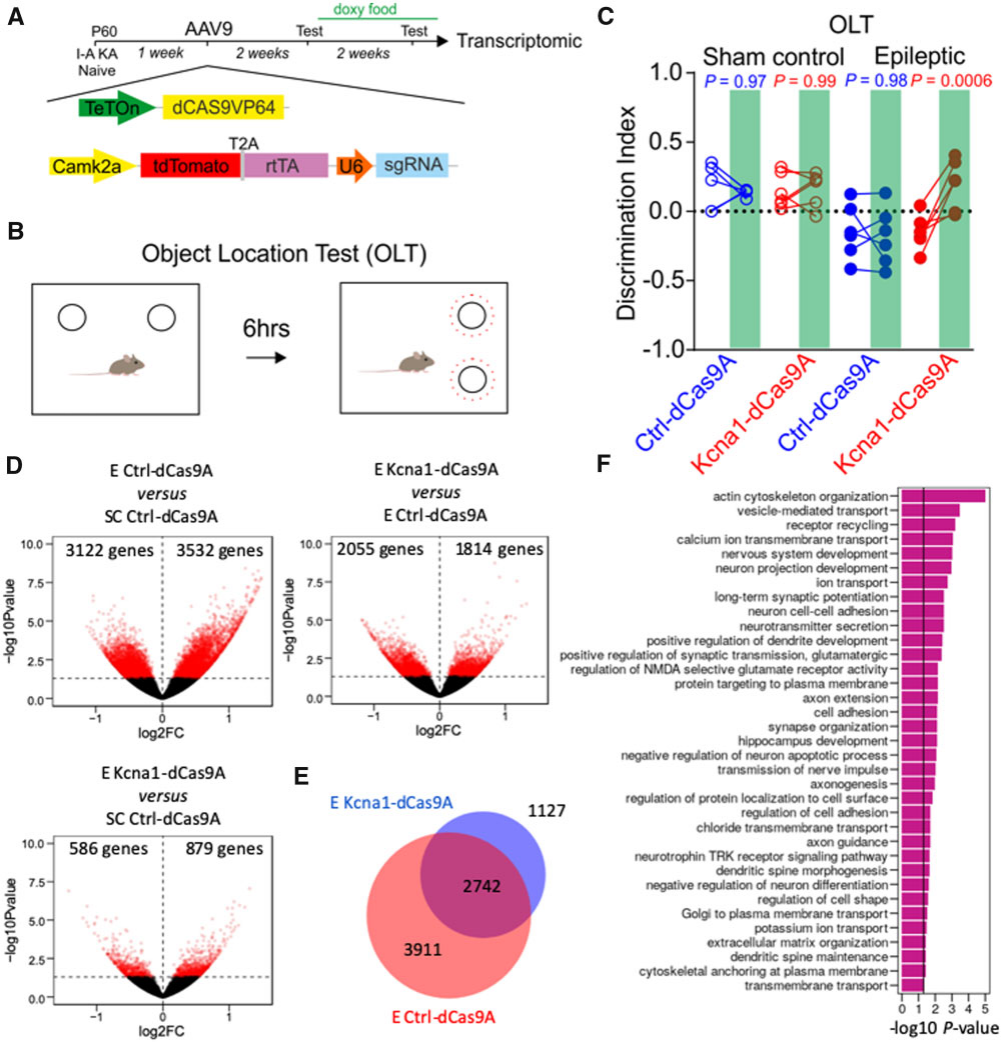
**CRISPRa-Kcna1 rescues cognitive deficits and mitigates transcriptomic changes in a mouse model of acquired intractable temporal lobe epilepsy.** (**A**) Experimental plan for behaviour and transcriptomic analysis. Mice were injected with AAVs a week after kainic acid (KA) treatment. Baseline behavioural tests were performed 2 weeks after AAV injection. Further behavioural tests were performed 2 weeks after doxycycline food supplementation. At the end of the experiment hippocampi were collected for transcriptomic analysis. (**B**) Graphical representation of the Object Location Test (OLT) and Novel Object Recognition Test (NORT). (**C**) Discrimination index for sham control and epileptic animals before and after (green box) doxycycline in mice treated either with Ctrl-dCas9A or Kcna1-dCas9A. Two-way ANOVA followed by Bonferroni multiple comparison test. (**D**) Volcano plots showing statistical significance (−log_10_*P-*value) as a function of fold-change in gene expression (log_2_FC), comparing pairs of datasets as indicated above each plot. Genes showing *P *<* *0.05 difference in expression (−log_10_*P-*value > 1.3) are highlighted in red. (**E**) Venn diagram showing the fraction of differentially regulated genes in epileptic Ctrl-dCas9A-treated mice (E Ctrl-dCas9A) which were rescued in Kcna1-dCas9A-treated mice (E Kcna1-dCas9A). (**F**) Histogram displaying representative gene ontology (GO) categories functionally enriched among the 2742 genes that were differentially expressed in epileptic control mice (E Ctrl-dCas9A) versus treated mice (E Kcna1-dCas9A).

Cognitive co-morbidities are an important feature of many forms of intractable epilepsy. We therefore asked if Kcna1-dCas9A treatment rescues behavioural deficits in our chronic intra-amygdala kainic acid model of temporal lobe epilepsy. We used two behavioural paradigms, one directly related to hippocampal function (Object Location Test) and the other more related to prefrontal cortex function (Novel Object Recognition Test) as previously described (Cho *et al.*, 2015). In the Object Location Test, no impairment in performance, as measured by the discrimination index, before and after doxycycline, were observed in sham control animals treated with either Ctrl-dCas9A or Kcna1-dCas9A (Fig. 4A–C). However epileptic mice showed a deficit compared to control mice (Ctrl-dCas9A sham control versus epileptic, *P *=* *0.01; Kcna1-dCas9A sham control versus epileptic, *P *=* *0.006; two-way ANOVA followed by Bonferroni multiple comparison test). This deficit was completely rescued only by Kcna1-dCas9A treatment (Fig. 4C). In contrast, no significant differences were observed in the Novel Object Recognition Test either between sham control and epileptic animals, or following treatment with either Ctrl-dCas9A or Kcna1-dCas9A (Supplementary Fig. 9) (Cho *et al.*, 2015). These data suggest that while Kcna1-dCas9A does not have adverse effects on hippocampal function in non-epileptic mice, it is able to restore function in epileptic mice.

Several studies have documented extensive changes in gene expression in different mouse seizure models (Motti *et al.*, 2010; Okamoto *et al.*, 2010; Winden *et al.*, 2011; Hansen *et al.*, 2014; Hawkins *et al.*, 2019). We asked if Kcna1-dCas9A was able to rescue transcriptomic changes in the temporal lobe epilepsy model by performing RNAseq analysis on hippocampi from sham control animals injected with Ctrl-dCas9A and epileptic animals injected either with Ctrl-dCas9A or with Kcna1-dCas9A. A comparison of gene expression between sham control (sham control Ctrl-dCas9A group) and epileptic animals (epileptic Ctrl-dCas9A group) revealed 6654 genes whose expression was altered (Fig. 4D). Kcna1-dCas9A treatment (epileptic Kcna1-dCas9A group) (Fig. 4E) rescued expression of 2742 of them (Fig. 4D and E). Consequently, the transcriptional profile of treated hippocampi (epileptic Kcna1-dCas9A group) was more similar to sham control mice (sham control Ctrl-dCas9A group), with only 1465 genes altered (Fig. 4D), than to epileptic Ctrl-dCas9A-treated mice. Sample distribution and K-means clustering along the first two principal components (PC1, PC2) failed to discriminate between sham control Ctrl-dCas9A and epileptic Kcna1-dCas9A groups, while the epileptic Ctrl-dCas9A group was distinct (Supplementary Fig. 10A). Gene ontology analysis of the 2742 genes rescued by Kcna1-dCas9A treatment (Fig. 4F) showed that it counteracted changes implicated in neurodegeneration and apoptosis (GO ID: negative regulation of anti-apoptotic process, hippocampus development, positive regulation of dendrite development, neuronal development with synapse formation, axon extension, axonogenesis, axon guidance). Several genes associated with neuronal activity were upregulated (GO ID: ion transport, calcium transmembrane, chloride transmembrane transport, potassium ion transport). The treatment also re-established expression of genes implicated in synapse function (GO ID: neurotransmitter secretion, synapse organization, dendritic spine maintenance, long term synaptic potentiation, transmission of nerve impulse, receptor recycling) and glutamatergic transmission (GO ID: positive regulation of glutamatergic synaptic transmission, regulation of NMDA selective glutamate receptor activity).

We compared the pattern of gene expression changes in our model with previously published studies (Motti *et al.*, 2010; Okamoto *et al.*, 2010; Winden *et al.*, 2011; Hansen *et al.*, 2014; Hawkins *et al.*, 2019); however, this failed to reveal a consistent signature across heterogeneous models of epilepsy (Supplementary Fig. 10B). Nevertheless, when restricting the comparison to focal kainic acid injection (Motti *et al.*, 2010), we identified 388 common deregulated genes, 165 of which were rescued in Kcna1-dCas9A treated mice (Supplementary Figs 10C, D and 11).

## Discussion

Although CRISPR has attracted intense interest as a possible treatment for inherited or acquired genetic disorders, it has, hitherto, received much less attention as a potential tool to treat acquired non-genetic diseases. The overwhelming majority of epilepsy cases, which represent an enormous disease burden, are not thought to be due to single gene mutations but are acquired during life, often secondary to a variety of brain insults such as infections, strokes and injuries (Kwan *et al.*, 2011; Tang *et al.*, 2017). Here we have shown that CRISPRa can be used to increase endogenous *Kcna1* expression to modulate neuronal activity, to reduce seizure initiation and to rescue behavioural and transcriptomic abnormalities in a mouse model of chronic temporal lobe epilepsy. This approach can potentially be used to regulate the expression of any gene, opening the way to treating many other neurological diseases associated with altered transcription.

At present, the main obstacles to clinical translation of the CRISPR/Cas9 toolbox are absence of long-term data on potential immunogenicity of the bacterial nuclease in humans and possible off-target effects that have not been detected by transcriptomic analysis (Kosicki *et al.*, 2018). Although in this study a non-mammalian nuclease was used, which can evoke an immune response in the primate CNS (Samaranch *et al.*, 2014), CNS disorders attributable to antibodies against nuclear neuronal proteins are rare, and rare forms of autoimmune encephalitis generally involve membrane proteins (Platt *et al.*, 2017). The doxycycline transactivator protein could also potentially evoke an immune response, although new inducible systems are in consideration for clinical translation (Das *et al.*, 2016; Kundert *et al.*, 2019). Cas9 has already been delivered with AAV in rodents and has been shown to induce a mild cellular response, but this has not been reported in the brain (Chew *et al.*, 2016). CRISPRa is, in principle, less likely to have deleterious off-target effects than CRISPR gene editing because it does not cleave DNA (La Russa and Qi, 2015; Zheng *et al.*, 2018), but further research is necessary.

Among distinct advantages of CRISPRa over exogenous gene delivery is the possibility to select one or more sgRNAs to tailor the exact level of gene expression independently by the number of viral copies effectively entered within each neuron. In addition, several sgRNAs could in principle be used in combination to control the transcription of heteromultimeric proteins such as GABA_A_ or NMDA receptors, or of multiple genes in a signalling pathway. Finally, whilst exogenous gene delivery is limited by the viral packaging capacity, CRISPRa can potentially be applied to control the activity of any gene irrespective of its length (Konermann *et al.*, 2015).

Although the present study made use of two AAVs to allow inducible activation of CRISPRa and expression of a fluorescent reporter protein, for clinical translation these features would not be required, as it should be possible to package both the dCas9 and the sgRNA in a single AAV to simplify clinical delivery. Further refinements can be considered, such as the use of an inducible promoter to allow the therapy to be switched off (Wykes and Lignani, 2018), which would not be possible with a gene editing strategy.

We have shown that is possible to upregulate an endogenous ion channel *in vitro* and *in vivo* and thereby to significantly change neuronal excitability (Figs 1 and 2). This result underlines an important paradigm of neuronal network, that relatively small changes in gene expression can robustly alter neuronal firing rates. We argue that these small changes in gene activation could be more sustainable long-term for a permanent modification of neuronal network activity without triggering adverse side effects such as protein accumulation or widespread transcriptomic compensation that could lead to less physiological alterations. Interestingly, the target gene upregulation was slightly lower when two sgRNAs were combined than when either of the sgRNAs was delivered on its own. This might be due to a relative reduction in transduction or transfection efficiency of each single sgRNA. We hypothesize that some cells/neurons received just one of the two sgRNAs and therefore the final output is a decrease in the total efficiency. This hypothesis is supported by the even smaller upregulation observed when three sgRNAs were combined together. A further unexpected finding was a decrease in input resistance observed in neurons transduced with Kcna1-dCas9A. This change was not observed with exogenous overexpression (Heeroma *et al.*, 2009; Wykes *et al.*, 2012) but potentially contributed to the therapeutic effect. Among possible explanations is that a homeostatic compensation by other channels was triggered by the increase of expression of the endogenous channel. We should, however, point out that Wykes *et al.* (2012) focused exclusively on layer 5 pyramidal neurons.

When moving to *in vivo* experiments we had to switch from lentivirus to AAV9 to transduce a larger area (whole hippocampus), which is not possible with lentivirus. We used an hSyn promoter *in vivo* to ensure specific expression in neurons and also used a dual-AAV system as SpdCas9 does not fit in a single AAV together with the sgRNA. For the same reason, we chose to use dCas9VP64 instead of VP160 used *in vitro*. Because of all these changes in the constructs, we tested the efficiency of the AAV9 in reducing neuronal firing *ex vivo*, showing a lower degree of firing reduction but still significantly lower than in control neurons (Fig. 2).

Importantly, in the present study we observed not only a decrease of seizure number but also the rescue of a behavioural co-morbidity and transcriptomic alterations that accompany epileptogenesis (Figs 3 and 4). Although the effect of our treatment on the absolute number of seizures was relatively modest, this is the first proof-of-principle that CRISPRa can be used *in vivo* to decrease spontaneous seizures. Thus far, this effect on seizures was demonstrated only in one model of chronic epilepsy. Further studies will be required to test other models of epilepsy before clinical translation. A single AAV system with a small dCAS9, possibly using multiple injection sites, such as in the contralateral hippocampus and amygdala, would potentially further improve the efficacy of suppressing seizures. Nevertheless, even the modest effect on number of seizures produced by this treatment was able to correct a behavioural co-morbidity. We tested the effects of epilepsy and dCas9A treatment in two behavioural tests previously studied in epileptic animals (Cho *et al.*, 2015), but several other tests, beyond the scope of this study, could be used to assess the extent of rescue of co-morbidities (Heinrichs and Seyfried, 2006).

Many studies have reported transcriptomic changes associated with epilepsy in different mouse models (Motti *et al.*, 2010; Okamoto *et al.*, 2010; Winden *et al.*, 2011; Hansen *et al.*, 2014; Hawkins *et al.*, 2019). However, no single gene alteration recurs across all available databases (Supplementary Fig. 9B). This inconsistency can be ascribed to differences in RNA-Seq technologies, epilepsy models, ages of animals, and delay after the insult. Our data add to the available data on gene expression, and provide, to our knowledge, the first evidence that gene therapy for epilepsy can correct dysregulation of a significant subset of genes.

Although we detected an upregulation of *Kcna1* in Kcna1-dCas9A injected hippocampus when compared to Ctrl-dCas9A injected tissue (Fig. 3C), we did not observe a clear increase in the expression levels of *Kcna1* using RNA-Seq (Supplementary Fig. 10). This may reflect a limitation of RNA-Seq due to a relatively low number of reads for *Kcna1*.

Importantly, the effect of CRISPRa-mediated *Kcna1* upregulation on generalized seizures may reflect an overall rescue of the pathology, including co-morbidities and gene expression, and not only an attenuation of acute seizures.

In conclusion, CRISPR-mediated control of gene expression can be successfully exploited to modulate neuronal activity and to mitigate seizures and behavioural co-morbidity in an experimental model of intractable temporal lobe epilepsy.

## Supplementary Material

awaa045_Supplementary_Materials

## Abbreviations

AAV =: adeno-associated virus
CRISPRa =: CRISPR activation
sgRNA =: small guide RNA
