# Supplementary material for: In vivo CRISPRa decreases seizures and rescues cognitive deficits in a rodent model of epilepsy
Source: Brain. 2020 Mar 4;143(3):891–905. doi: 10.1093/brain/awaa045 (PMC7089667; doi:10.1093/brain/awaa045)
Supplement: awaa045_Supplementary_Materials [file awaa045_supplementary_materials.zip › awaa045-suppl_data/Supplementary Materials.pdf]

Supplementary Materials

Table 1. sgRNA and primer sequences

Name	Sequence
sglacZ	TGCGAATACGCCCACGCGAT
Sg4	CTTCGCACCACCGCGGCTCG CGG
Sg14	GGGGCCTCTAGAAGGATCCC AGG
Sg19	AGTCAATGATCACATCCTCC TGG
Sg30	ACCCCAGCATTCCTTTCAGG GGG
18s_F	GGTGAAATTCTTGGACCGGC
18s_R	GACTTTGGTTTCCCGGAAGC
Kcna1_F	TTCAGACTCTCCGCCGACTC
Kcna1_R	CAGGCCTGTCACCCACTTTG
dCas9-VP64_F	TCCTTTTTGGTGGAGGAGGA
dCas9-VP64_R	TCAACCGCAAGTCAGCCTTA
Pde4b_F	TCAGCCAGGTCTAATCTGCCA
Pde4b_R	ACCGCCATCACACTCCTACT
Mylpf_F	CTCACCTCCCCGAATGTCCT
Mylpf_R	GGGTCCCCTATTCCTGGTCC

Efcab4a_F	CCAGCATTCCACCATTCCCC
Efcab4a_R	GTCGACTGAGCTGCTCTTGG
Nudcd2_F	CCGAGCCCTAAAATTCACGGT
Nudcd2_R	ACAGTTTCCCCTTGCCACAC
Gc_F	AGGAGGTGCTGCAAGACTCT
Gc_R	CCTTCTCATAGTCTCGGCCTCT
Vps16_F	ACACTGCGAACTGGAATCCAC
Vps16_R	GCAATCCTTGAGCTCCTCCTTC

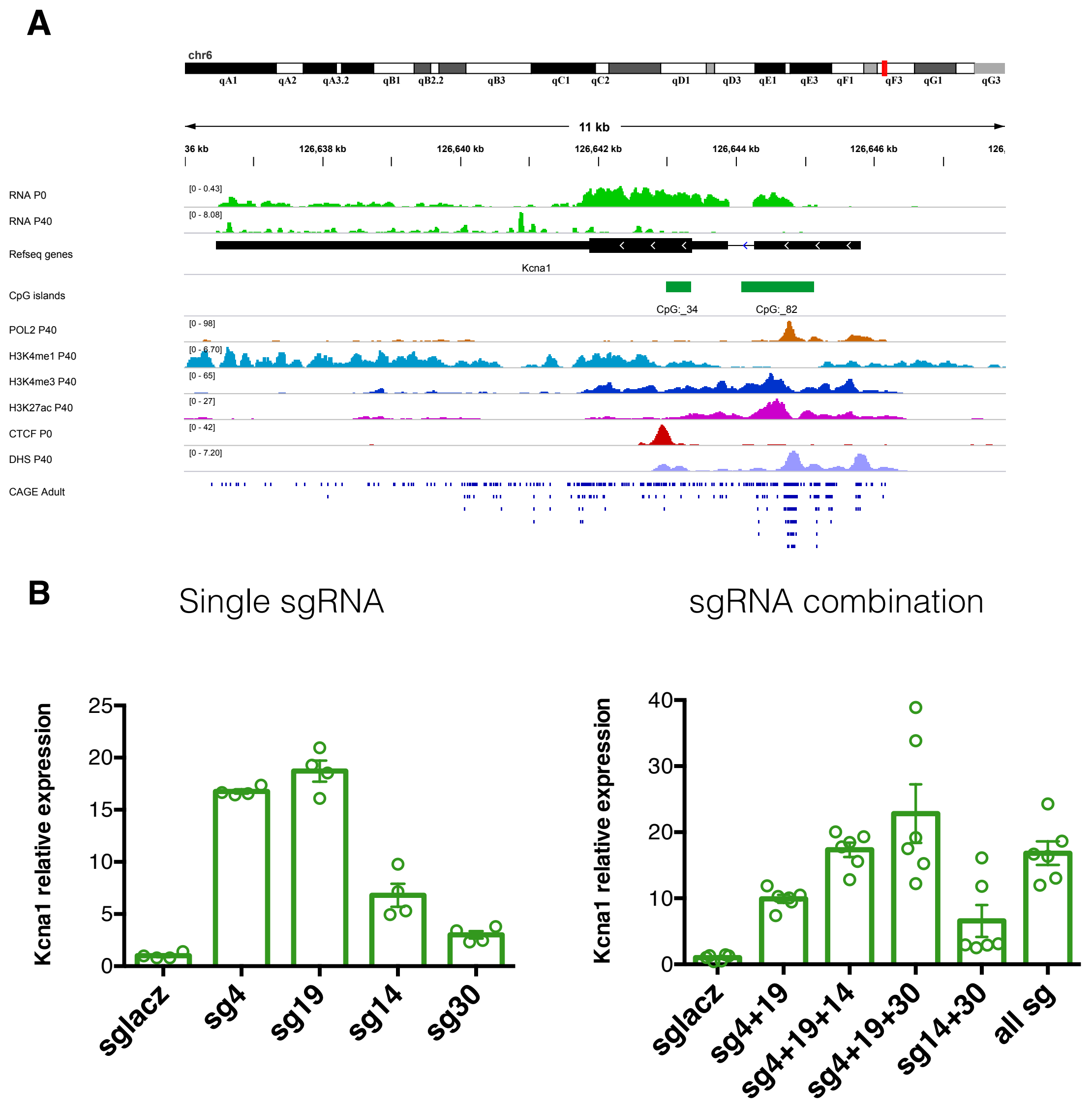

Figure S1: Bioinformatics analysis for prediction of *Kcna1* gene promoter and sgRNA screening for stimulating *Kcna1* gene expression with CRISPRa in P19 cells. A. Alignment of *Kcna1* gene reference sequence with RNA-seq of mouse brain at P0 and P40, ChIP-seq at P40 for POL2 (RNA polymerase II), H3K4me1 (mono-methylation of lysine 4 on histone H3), H3K4me3 (tri-methylation of lysine 4 on the histone H3) and H3K27ac (acetylation of lysine 27 on histone H3), CTCF (factor that binds the CCCTC) and DNase-seq (DHS, DNase I Hyper Sensitivity mapping) and CAGE-seq (Cap Analysis of Gene Expression-sequencing) profiles. The enrichment of markers associated with transcriptional activation in the regions upstream of the first exon of the gene highlights the presence of a TSS and points to a promoter region

200 bp upstream. **B**. RT-qPCRs for *Kcna1* mRNA levels on RNA extracted from P19 cells lipofected with dCas9VP160-T2A-Puro^R together with sgRNAs targeting Kcna1 gene promoter. Data are normalized by 18S rRNA and are shown relative to control sgLacZ cells.

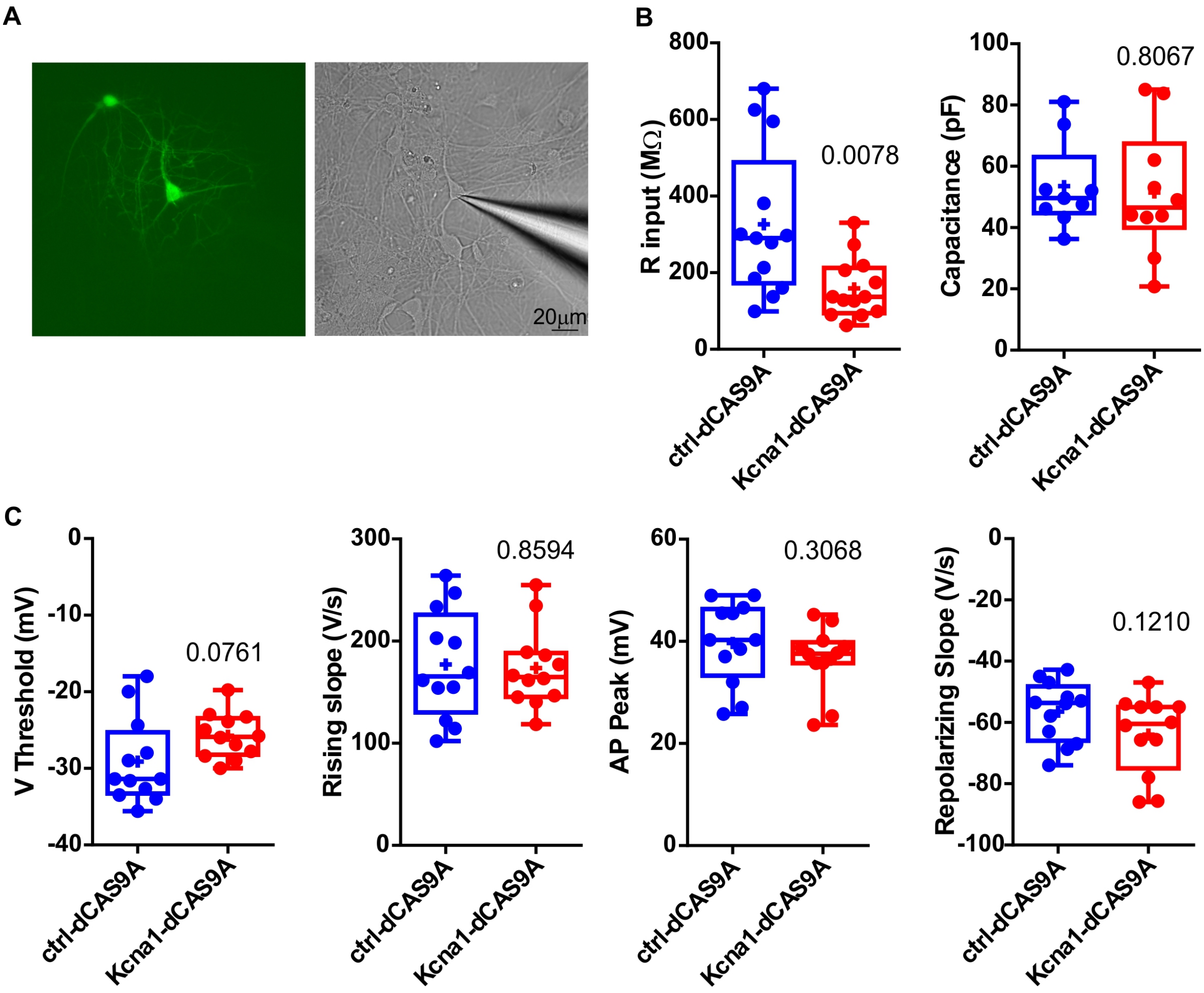

Figure S2: Electrophysiology recordings from cultured neurons. Neuronal and AP parameters. A. representative picture of a patched transduced neuron in vitro (Green, EGFP). **B, C.** Passive (B) and active (C) parameters of recorded cells. Student's t test. Only the input resistance was significantly different beetween Ctrl-dCas9A and Kcna1-dCas9A neurons.

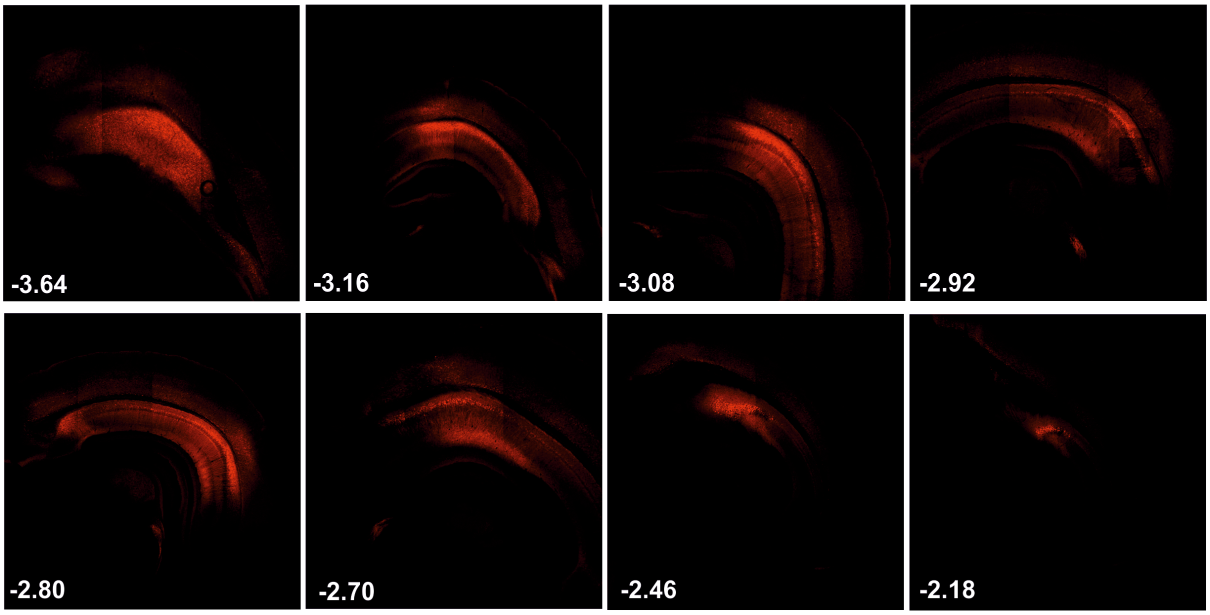

Figure S3: tdTomato expression in a Camk2a/CRE mouse. Representative images from a Camk2a/CRE mouse injected with Ctrl-dCas9A. Native tdTomato (non-immunofluorescence) was present in the floxed rtTa-t2a-tdTomato cassette driven by Syn promoter. Coordinates are from Bregma.

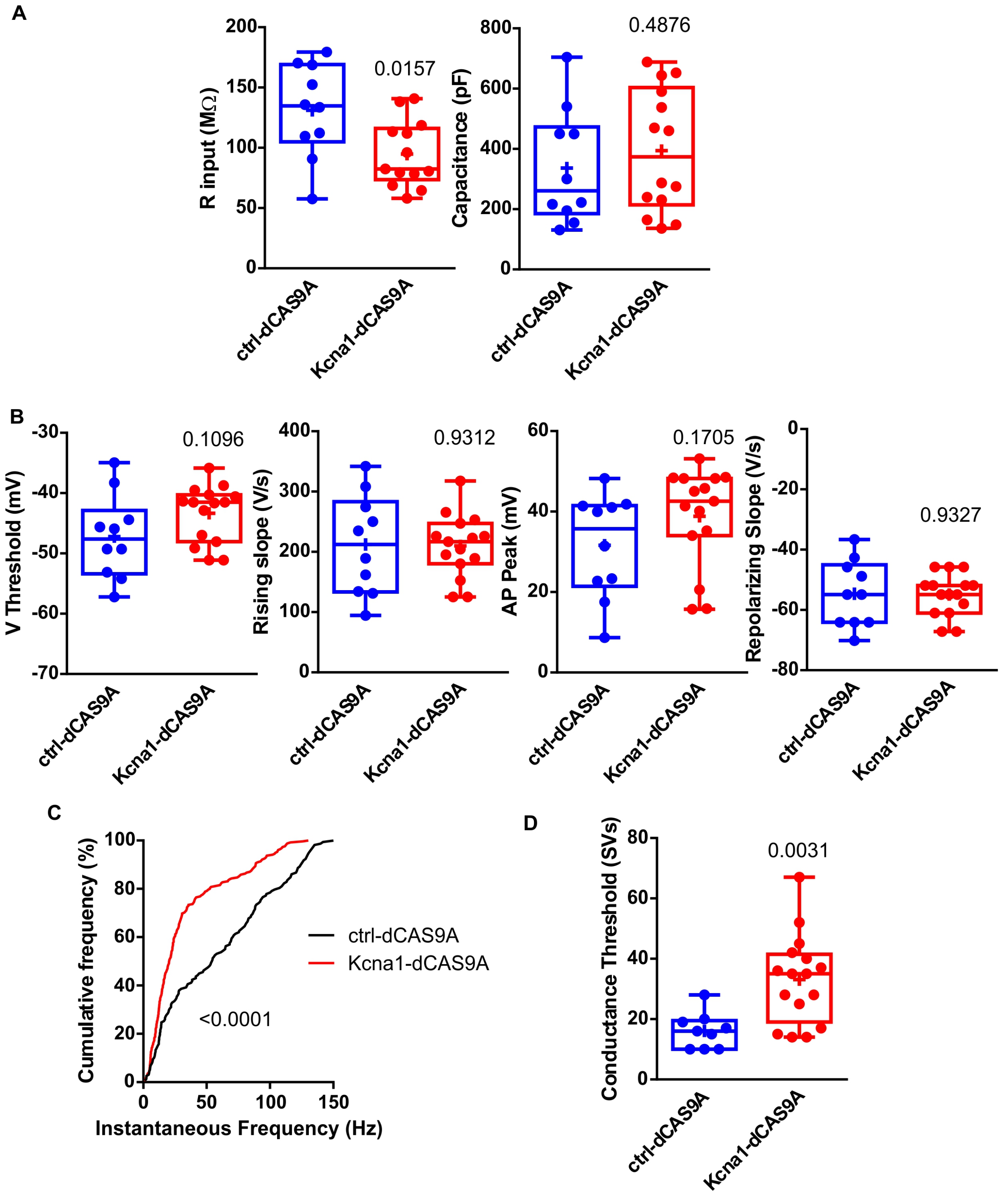

Figure S4: ***Ex vivo*** **electrophysiological recordings in acute slices from Camk2a/CRE mice. A, B.** Passive (B) and active (C) parameters. Student's t test. Only the input resistance was significantly different between Ctrl-dCs9A and Kcna1-dCs9A neurons. **C.** Cumulative analysis of instantaneous frequency for the first 2 APs in each current step for all neurons transduced with either Ctrl-dCas9A or Kcna1-dCs9A. Mann-Whitney non-parametric test. **D.**

Conductance threshold calculated as the first AP elicited with steps of simulated single AMPA miniature events, SVs is defined as number of synaptic vesicles deducted from single miniature events (Morris *et al.*, 2017).

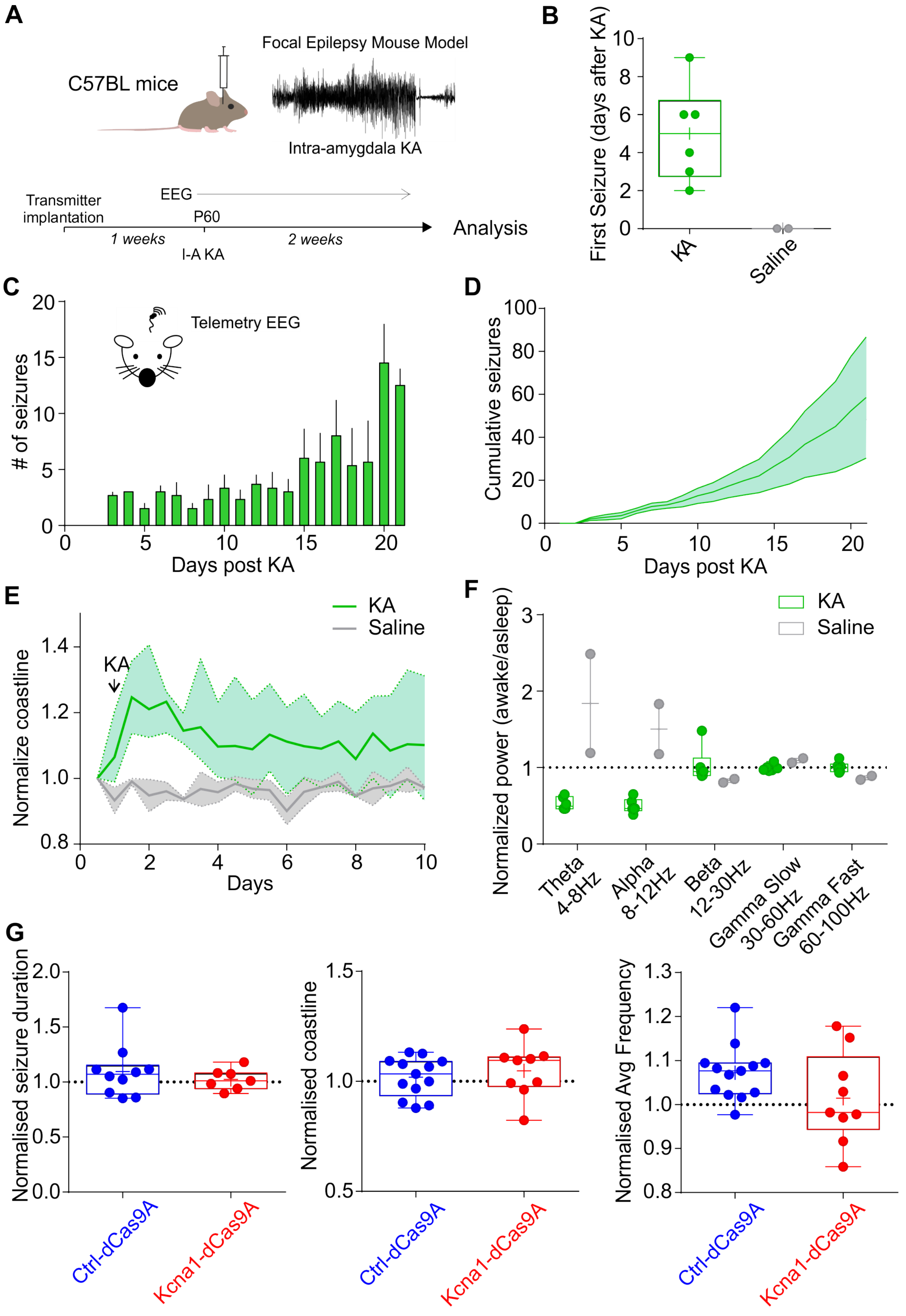

Figure S5: Intra-amygdala kainic acid model of temporal lobe epilepsy. A. Experimental plan for inducing chronic epilepsy by injecting KA in the right amygdala to induce status

epilepticus (SE). **B.** Latency from KA to the first stage 5 generalized tonic-clonic seizure in animals injected with KA or saline. The first seizure occurred, on average, 5 days after KA. **C, D.** Number of seizures (C) and cumulative number of seizures (D) in the first 3 weeks after KA injection (n=6). **E.** Coastline in the first 10 days after SE normalized to 12hr recordings before SE for animals injected with KA (n=6) or saline (n=2). Data points are binned every 12hrs. **F.** Power during wakefulness (defined as the 12hr dark period) as a ratio of power during sleep (defined as the 12hr light period) for the theta, alpha, beta, slow and fast gamma bands. **G.** Seizure duration, coastline and power in the different bands recorded after doxycycline, normalized by values before doxycycline, did not differ between groups. The normalized averaged frequency was calculated as the power-weighted frequency across all frequency bands.

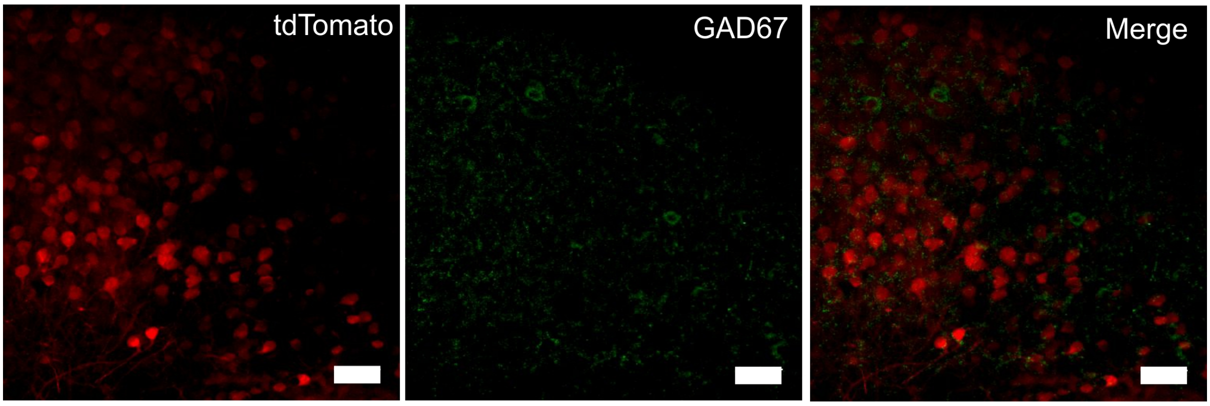

Figure S6: Immunohistochemistry of epileptic brains after the recordings. Representative image of a Ctrl-dCas9A injected mouse showing no co-localization between tdTomato, driven by Camk2a promoter, and GAD67, a marker for inhibitory neurons (scale bar: 50µm). No co-localisation between Camk2a-driven tdTomato and GAD67 was detected in any of the 4 brains analysed after recordings.

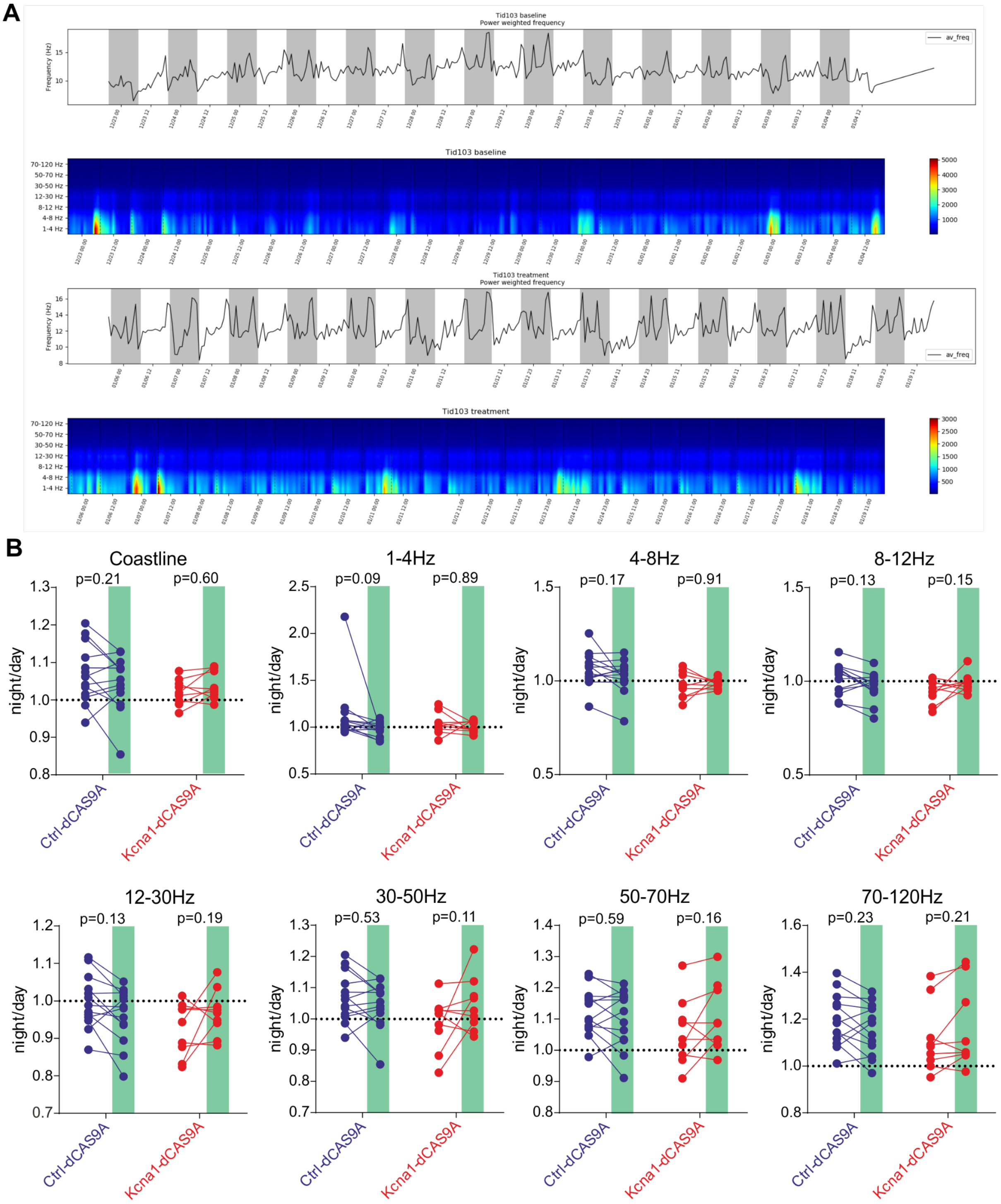

Figure S7. CRISPRa-Kcna1 did not differentially affect EEG power during night or day, in epileptic mice. A. Representative spectrogram of total frequency and of individual frequencies before (top panels) and after doxycycline (bottom panels) **B.** Quantification of coastline and EEG power at different frequencies as ratio between night and day. No effect of

doxycycline was observed in control- and Kcna1-dCas9A treated animals. Two-way ANOVA followed by Bonferroni multiple comparison test.

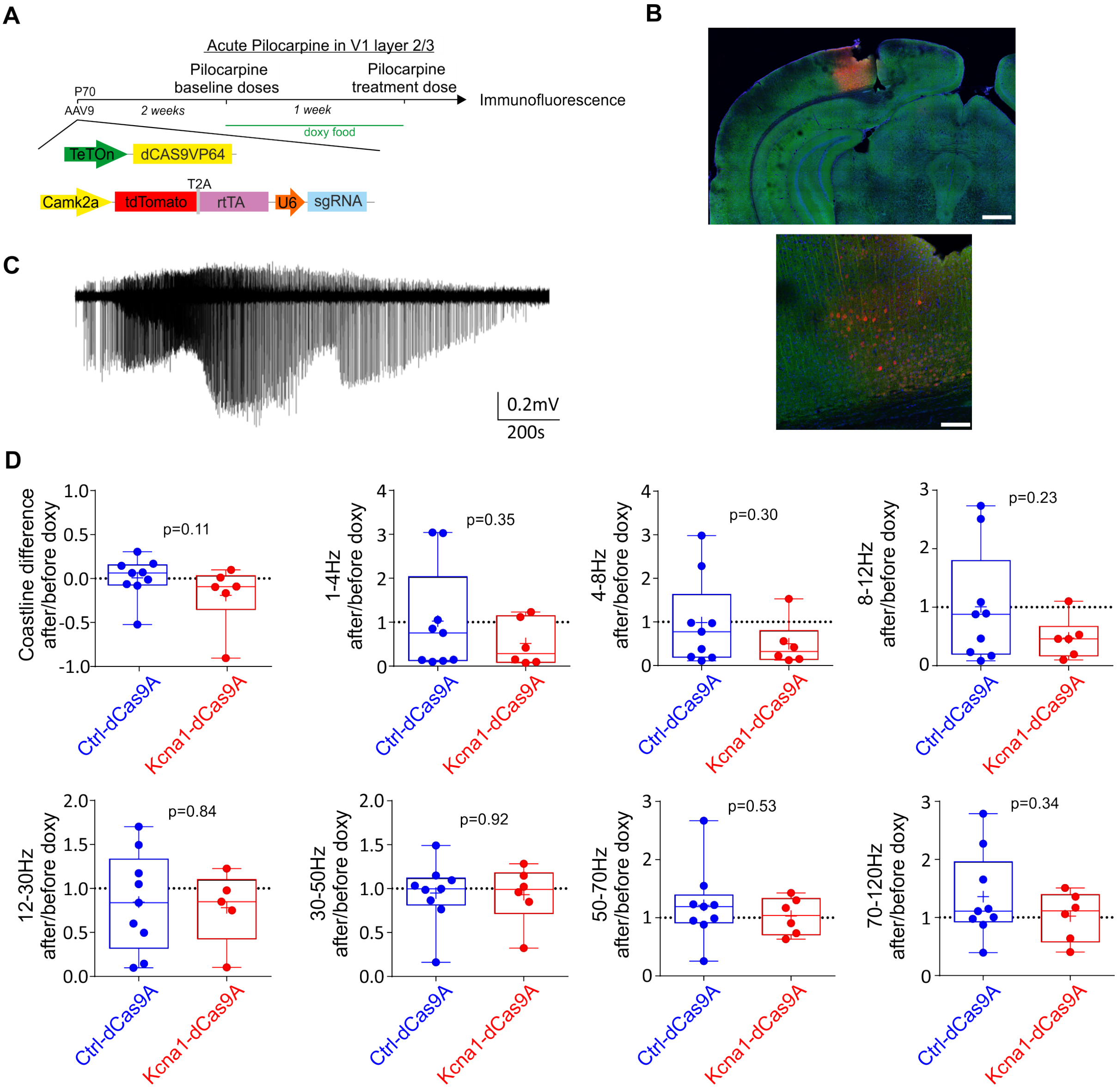

Figure S8: Acute pilocarpine injection. A. Graphical experimental design (see Material and methods). **B.** Representative coronal image from a Ctrl-dCas9A injected mouse at the site of injection, with transduced neurons in red, MAP2 in green and DAPI in blue. Scale bar top: 0.5mm; bottom: 0.1mm. **C.** Representative acute seizure induced by pilocarpine. **D.** Seizure parameters after and before doxycycline administration. Coastline measured either before or after doxycycline was normalized by a period before pilocarpine injection, and is shown as the difference (after – before doxycycline). Student's t test.

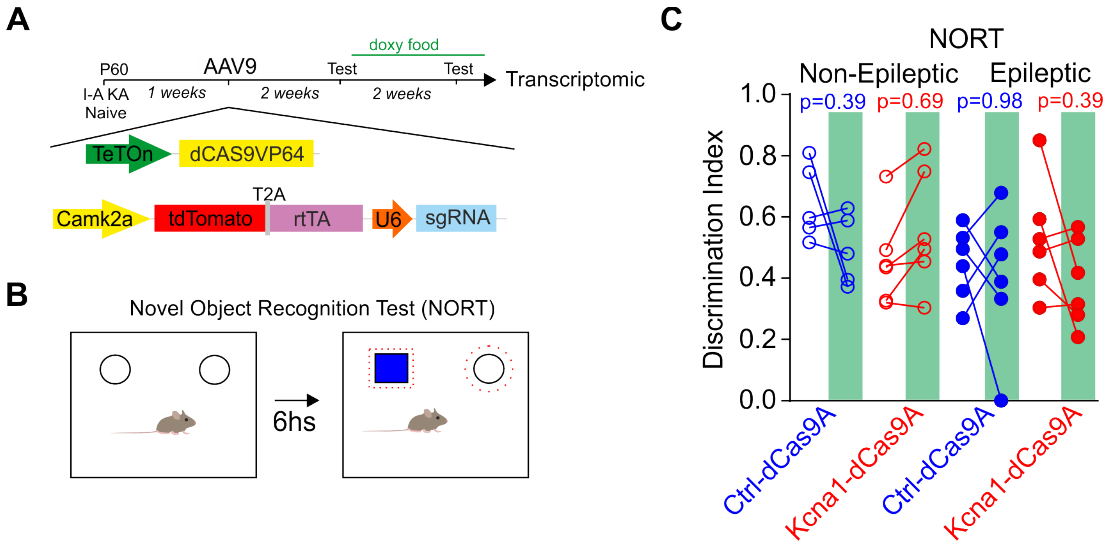

Figure S9: CRISPRa-Kcna1 did not change behaviour in the novel object recognition test (NORT). A. Experimental plan. **B**. Graphical representation of NORT. **C**. Discrimination index for non-epileptic and epileptic animals before and after (green box) doxycycline in mice treated either with Ctrl-dCas9A or Kcna1-dCas9A. Two-way ANOVA followed by Bonferroni multiple comparison test.

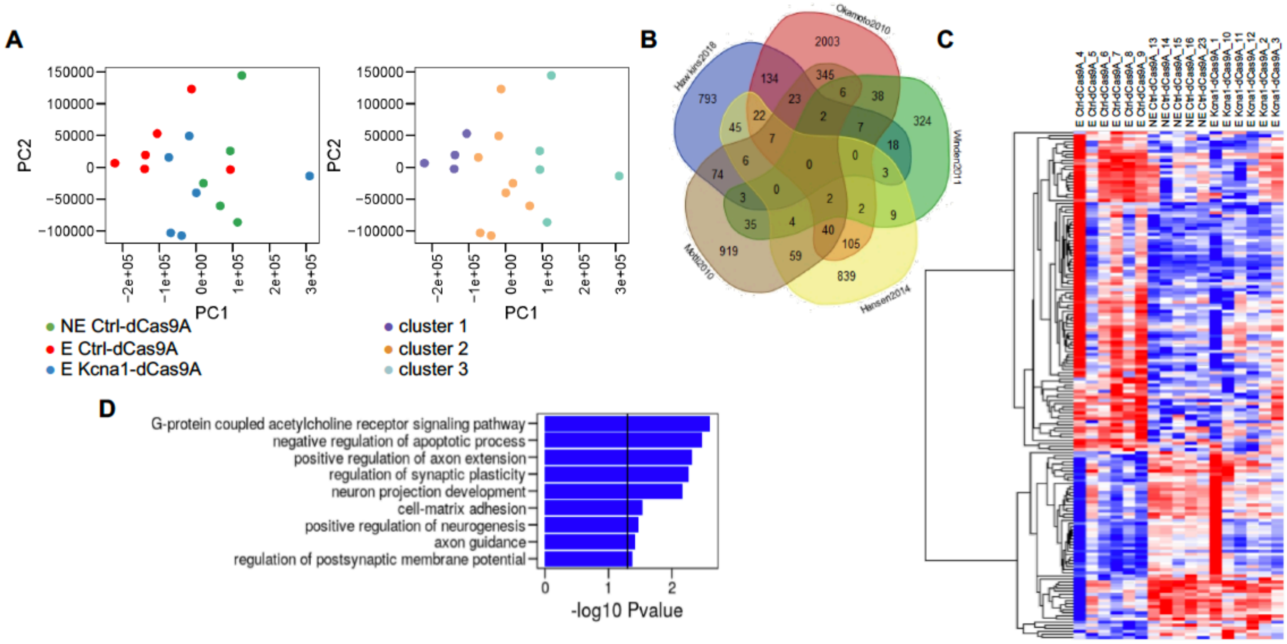

Figure S10: **Changes in gene transcription in the epilepsy model**. **A**. Sample distribution along principal components one (PC1) and two (PC2) from whole transcriptome Principal Component Analysis (PCA), color-coded according to sample group or K-Means machine learning clustering. **B**. Venn diagram displaying the genes reported as differentially expressed in genetic (Scn1a +/-) and pharmacologically induced (Kainate, Pilocarpine) murine epilepsy models, with the source literature indicated.

C. Gene expression heatmap showing 165 dysregulated genes in both kainate-treated epileptic mice (Motti et al., 2010) and this study, and partial rescue with the Kcna1-dCas9A treatment.

D. Bar chart showing gene ontology (GO) categories that are functionally enriched among the aforementioned 165 genes.

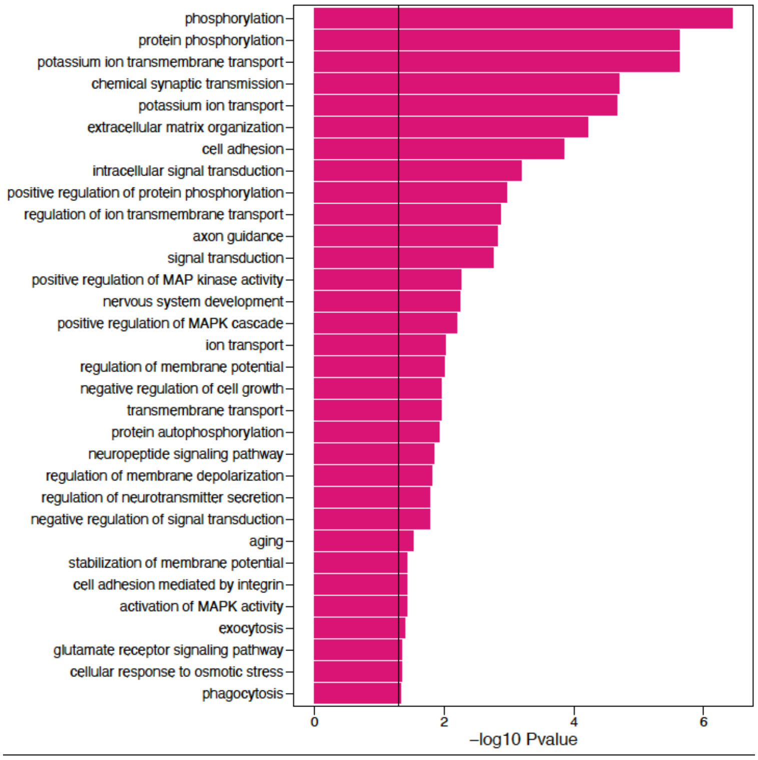

Figure S11: Gene ontology categories not rescued by the treatment. Comparison between non-epileptic Control vs Epileptic dCas9-Kcna1.

Video S1: Representative video of SE in a C57BL/6J mouse (2 minutes from a 40 minute-long episode of SE).

Video S2: Representative video of a Stage 5 generalized tonic-clonic seizure in a C57Bl/6J mouse 4 weeks after KA injection and transduced with ctrl-dCAS9A.
