# Supplementary figures and images for: In vivo CRISPRa decreases seizures and rescues cognitive deficits in a rodent model of epilepsy
Source: Brain. 2020 Mar 4;143(3):891–905. doi: 10.1093/brain/awaa045 (PMC7089667; doi:10.1093/brain/awaa045)

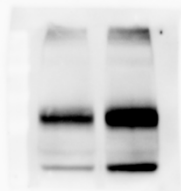

Supplement: awaa045_Supplementary_Materials [file awaa045_supplementary_materials.zip › awaa045-suppl_data/Supplementary Fig 2.pdf]

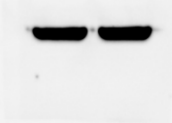

Supplement: awaa045_Supplementary_Materials [file awaa045_supplementary_materials.zip › awaa045-suppl_data/Supplementary Fig.pdf]
